# Immobilization of Lewis Basic Nitrogen Sites into a Chemically Stable Metal–Organic Framework for Benchmark Water‐Sorption‐Driven Heat Allocations

**DOI:** 10.1002/advs.202105556

**Published:** 2022-02-11

**Authors:** Bin Li, Feng‐Fan Lu, Xiao‐Wen Gu, Kai Shao, Enyu Wu, Guodong Qian

**Affiliations:** ^1^ State Key Laboratory of Silicon Materials School of Materials Science and Engineering Zhejiang University Hangzhou 310027 China

**Keywords:** chillers, coefficient of performances, heat pump, porous materials, water adsorption

## Abstract

Developing efficient and stable water adsorbents for adsorption‐driven heat transfer technology still remains a challenge due to the lack of efficient strategies to enhance low‐pressure water uptakes. The authors herein demonstrate that the immobilization of Lewis basic nitrogen sites into metal–organic frameworks (MOFs) can improve water uptake and target benchmark coefficient of performances (COPs) for cooling and heating. They present the water sorption properties of a chemically stable MOF (termed as Zr‐adip), designed by incorporating hydrophilic nitrogen sites into the adsorbent MIP‐200. Zr‐adip exhibits S‐shaped sorption isotherms with an extremely high water uptake of 0.43 g g^−1^ at 303 K and *P*/*P*_0_ = 0.25, higher than MIP‐200 (0.39 g g^−1^), KMF‐1 (0.39 g g^−1^) and MOF‐303 (0.38 g g^−1^). Theoretical calculations reveal that the incorporated N sites can serve as secondary adsorption sites to moderately interact with water, providing more binding sites to strengthen the water binding affinity. Zr‐adip achieves exceptionally high COPs of 0.79 (cooling) and 1.75 (heating) with a low driving temperature of 70 °C, outperforming MIP‐200 (0.78 and 1.53) and KMF‐1 (0.75 and 1.74). Combined with its ultrahigh stability, excellent cycling performance, and easy regeneration, Zr‐adip represents one of the best water adsorbents for adsorption‐driven cooling and heating.

## Graphical abstract

The immobilization of Lewis basic nitrogen sites into metal–organic frameworks has been demonstrated to be capable of improving water uptake at low relative pressure, in which a novel stable Zr‐adip exhibits an ultrahigh water uptake of 0.43 g g^−1^ at 303 K and 25% RH, thus affording the benchmark COP_C_ and COP_H_ values under an ultralow driving temperature of 70 °C.

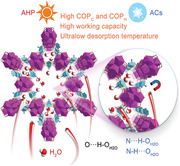

## 1 Introduction

Global energy consumption of heating and cooling for both industrial and residential purposes has increased gradually and is expected to rapidly raise in the coming years.^[^ ^1^, ^2^ ^]^ Current systems used in heating and cooling applications mainly depend on non‐sustainable energy resources derived from fossil fuels, and mostly consist of compressors employing harmful chlorofluorocarbons (CFCs) and partially hydrogenated CFCs as working fluid.^[^ ^3^, ^4^, ^5^ ^]^ These energy and environment problems have pushed the development of sustainable and more energy‐efficient systems implying clean and renewable energy resources to be of utmost importance. In this regard, green adsorption‐driven heat pumps (AHP) and chillers (AC) using water as working fluid have attached great interest due to the potential use of low‐grade and environmentally sensitive heat sources (e.g., industrial waste heat or solar energy). Such a technology typically features a two‐step process consisting of a working cycle and a regeneration cycle that accompanies with a full cycle of water adsorption/desorption (Figure S1, Supporting Information).^[^ ^6^, ^7^, ^8^, ^9^ ^]^ The efficiency of this technology depends highly on the amount of water that can be exchanged between the adsorption and desorption stages.

Water adsorbents play a vital role in determining the efficiency of AC and AHP systems. An increase of uptake capacity at relative low pressures and a decrease of driving temperature are both important to enhance the heat transfer efficiency. Traditional lithium chloride (LiCl), silica gel, and zeolites have been widely used as commercial water adsorbents. However, these materials suffer from clear drawbacks, including the risk of toxicity and corrosion (in the case of LiCl/H_2_O working pair),^[^ ^10^, ^11^ ^]^ weak hydrophilicity to result in insufficient adsorption capacity at low relative pressures (e.g., silica gel), and ultra‐strong hydrophilic character that requires very high regeneration temperatures above 150 °C (e.g., hydrophilic zeolites).^[^ ^12^ ^]^ At present, the CHA‐type silico‐aluminosilicate SAPO‐34 has appeared as a commercially usable water adsorbent for adsorption‐driven chillers because of its relatively high uptake capacity at low pressure (*P*/*P*_0_ < 0.1) and very high durability and robustness.^[^ ^13^ ^]^ Nevertheless, the regeneration temperature of this material (90 °C) is incompatible with the desired use of low‐temperature heat sources, and its low working capacity (0.20 g g^−1^ at 95 °C) is a great limitation to the performance of cooling and heating applications.

There is nowadays a tremendous interest to develop novel porous materials able to achieve high water uptake at low relative pressures (*P*/*P*_0_ = 0.1–0.3) so as to target high AC and AHP performance. In this regard, porous metal–organic frameworks (MOFs) have received immense attention as promising water adsorbents owing to their powerful predictability and tunability on pore size/shape and functionality.^[^ ^14^, ^15^, ^16^, ^17^, ^18^, ^19^, ^20^, ^21^, ^22^, ^23^, ^24^, ^25^ ^]^ Reticular chemistry of MOFs has enabled us to design and control of pore size, surface area, and hydrophilicity at the molecular level.^[^ ^26^, ^27^, ^28^, ^29^, ^30^, ^31^, ^32^, ^33^, ^34^, ^35^, ^36^ ^]^ In the past two decades, a number of MOFs have been developed as water adsorbents for diverse water adsorption‐related applications.^[^ ^37^, ^38^, ^39^, ^40^, ^41^, ^42^, ^43^, ^44^, ^45^, ^46^, ^47^, ^48^, ^49^ ^]^ Regarding AC and AHP applications, the design of a viable water adsorbent would meet the following four criteria: 1) steep uptake behavior at low relative pressure (*P*/*P*_0_ < 0.3) with high working capacity; 2) high coefficient of performance (COP) and facile water adsorption/desorption kinetics for energy efficiency; 3) regeneration at low temperature (*T* < 70–80 °C); 4) excellent chemical stability and cycling performance. While many stable MOFs have been developed as water adsorbents, it still remains a daunting challenge to design ideal adsorbents to meet the above criteria. For example, some large‐pore MOFs (e.g., MIL‐101, Cr‐soc‐MOF‐1, and MOF‐808) exhibit large water uptake that is typically reached only at high relative pressure; however, the weak affinities for water make their steep uptake behavior occur at high relative pressure, which is unfavorable to AC and AHP applications.^[^ ^37^, ^50^, ^51^, ^52^, ^53^ ^]^ On the other hand, while those MOFs with small pores can capture water at low *P*/*P*_0_ values (<0.3) with steep uptake behavior, their low pore volumes delimit the total water uptakes and thus working capacity, as exemplified by CAU‐10 and CAU‐23.^[^ ^54^, ^55^, ^56^, ^57^, ^58^ ^]^ To improve water adsorption at low relative pressures, the incorporation of hydrophilic functional sites into porous MOFs (e.g., open metal sites, —OH, and —NH_2_) has been documented to be a feasible strategy to increase pore hydrophilicity and polarity.^[^ ^59^, ^60^, ^61^, ^62^ ^]^ The well‐established cases of MOFs are MOF‐74 with open metal sites that can strongly bind with water, resulting in ultrahigh water uptakes at *P*/*P*_0_ < 0.1 and also high regeneration temperatures (over 190 °C).^[^ ^31^, ^63^, ^64^, ^65^ ^]^ Further, as typically observed in the —OH and —NH_2_‐functionalized CAU‐10 and MIL‐101(Cr), the desired higher affinity for water was attained at the cost of a significant drop in uptake capacity, since the incorporated bulky functional sites lead to a notable decrease in pore volume of the materials.^[^ ^66^, ^67^, ^68^ ^]^

To overcome the above limitations, it is of profound importance to develop an alternative strategy that can efficiently improve water affinity while without sacrificing low regeneration temperature and moderate pore volumes or surface areas to take up large amount of water molecules at low *P*/*P*_0_ values. In this context, we speculated that the immobilization of Lewis basic nitrogen atoms to replace carbon atoms into the organic linkers of MOFs may provide great potential to target this matter in view of the following considerations. First, nitrogen substitution would not induce the reduction on surface area or pore volume since the size of nitrogen atom is comparable to carbon. Second, the incorporation of N sites can provide additional binding sites to form weak hydrogen‐bonding with water molecules, improving pore hydrophilicity and water uptake capacity at low relative pressures. Third, unlike MOF‐74 with strong OMSs,^[^ ^65^ ^]^ such weak hydrogen‐bonding interactions would not result in the obvious increase on water uptake at *P*/*P*_0_ < 0.1 or regeneration energy, thus maximizing the working capacity for these applications.

With these merits in mind, we herein report the incorporation of Lewis basic N sites into stable MOFs for highly boosting water uptake capacities at *P*/*P*_0_ < 0.25 and thus promoting the COP values for AC and AHP applications. Through a comprehensive screening of the reported water‐sorption MOFs, we elaborately selected a highly stable Zr‐MOF (MIP‐200 reported by Serre and colleagues) as the fundamental framework backbone to the nitrogen functionalization because of its suitable pore sizes, high water uptake at *P*/*P*_0_ < 0.25, high stability, and excellent COP values.^[^ ^69^ ^]^ To target this matter, we designed and synthesized a new ligand of 5,5′‐azanediyldiisophthalic acid (H_4_adip) by using an —NH— polar spacer to replace —CH_2_— group in the linker of MIP‐200, and used it to construct a novel isoreticular framework (Zr‐adip). As expected, the immobilized —NH— sites in Zr‐adip lead to an enhanced pore hydrophilicity meanwhile maintain its higher pore volume compared with MIP‐200. Zr‐adip thus features typically S‐shaped water sorption isotherms with notably improved uptake capacities in a low relative pressure range (*P*/*P*_0_ = 0.10–0.25). Most importantly, this material exhibits an ultrahigh water uptake of 0.43 g g^−1^ at 303 K and *P*/*P*_0_ = 0.25, higher than that of MIP‐200 (0.39 g g^−1^)^[^ ^69^ ^]^ and all the other benchmark materials including SAPO‐34 (0.28 g g^−1^),^[^ ^13^ ^]^ KMF‐1 (0.39 g g^−1^),^[^ ^70^ ^]^ and MOF‐303 (0.38 g g^−1^).^[^ ^71^ ^]^ Theoretical calculations revealed that the incorporated —NH— sites can serve as secondary adsorption sites to moderately interact with water molecules, thus improving the water uptake capacities at *P*/*P*_0_ = 0.10–0.25. This material thus achieves exceptionally high COP values of 0.79 (cooling) and 1.75 (heating) along with high volumetric working capacities under a low driving temperature of 70 °C, both of which outperform those of MIP‐200 (0.78 and 1.53) and KMF‐1 (0.75 and 1.74).^[^ ^69^, ^70^ ^]^

## 2 Results and Discussion

### 2.1 Synthesis and Characterization of Zr‐adip

The organic linker H_4_adip was readily synthesized through a two‐step reaction procedure (Scheme S1, Supporting Information), and its purity was confirmed by ^1^H NMR spectroscopy (Figure S2, Supporting Information). The reaction of H_4_adip ligand with ZrCl_4_ in a mixture of acetic anhydride and formic acid at 393 K for 48 h afforded a white powder sample of Zr‐adip with a high yield and high crystallinity. Such acetic anhydride and formic acid mixture solvents are considered as green solvents with a much lower toxicity than dimethylformamide (DMF) and dimethylacetamide, indicating a more environmentally friendly synthesis condition. Despite extensive attempts, we were not able to obtain large single crystals of Zr‐adip for single‐crystal X‐ray diffraction studies. The powder X‐ray diffraction (PXRD) patterns indicate that Zr‐adip expectedly has a similar structure to MIP‐200 (Figure S3, Supporting Information). In addition, the phase purity and high crystallinity of bulk Zr‐adip were further confirmed by the PXRD.

**Figure 1:**
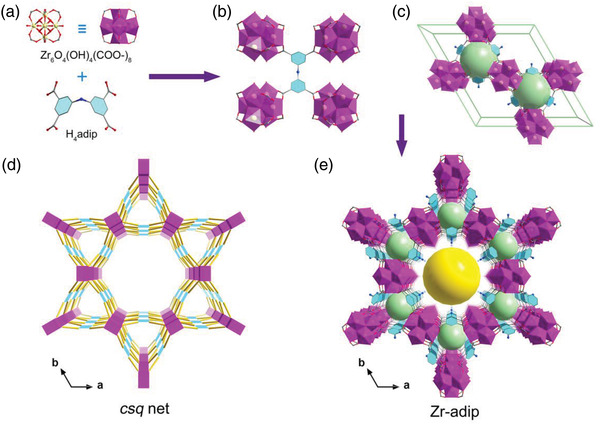
Crystal structure of Zr‐adip. a) The 8‐connected Zr_6_O_4_(OH)_4_(COO^–^)_8_ SBUs and the H_4_adip ligand. b) One H_4_adip molecule bonded to four SBUs. c) *csq* topological network of the MOF structure. d) The *csq* topology of Zr‐adip. e) The hexagonal and triangular channels with the diameter of 6.6 and 12.8 Å, respectively, viewed along the *c*‐axis. Color code: Zr (purple), O (red), C (grey), N (cyaneous); the H atom is omitted for clarity.

We relied on synchrotron X‐ray diffraction (SXRD) to determine the crystal structure of Zr‐adip. Synchrotron diffraction data was collected by BL14B1 at the Shanghai Synchrotron Radiation Facility with microfocused X‐rays. Through indexing of the SXRD data, a hexagonal *P*6/*mmm* space group was first identified for the Zr‐adip crystal. We thus modeled its structure using the same framework connection as MIP‐200. Based on the structure model, Rietveld refinement was performed on the SXRD data, and we obtained the unit cell parameters of *a* = *b* = 25.5265 Å and *c* = 11.5812 Å with agreement factors of *R*_p_ = 0.0384 and *R*_wp_ = 0.0680 for Zr‐adip (Figure S4, Supporting Information), strongly supporting its validity. Detailed structure information of Zr‐adip is provided in Tables S1 and S2, Supporting Information. Crystal structural analysis revealed that Zr‐adip adopts a 3D network with the same cube‐and‐square (*csq*) topology to MIP‐200. As shown in **Figure** **1**, the network of Zr‐adip is composed of Zr_6_O_4_(OH)_4_ secondary building units (SBUs) interconnected by eight adip linkers, and each of the linkers is coordinated to four SBUs. In addition, the coordination of the SBU might be completed with terminal formate ligands, which can be replaced by hydroxyl groups or water molecules under treatment with concentrated HCl, as demonstrated in the isoreticular MIP‐200.^[^ ^69^ ^]^ As depicted in Figure 1e, Zr‐adip shows two types of 1D pore channels along the *c* axis: one small triangular channel with a diameter of 6.6 Å and another large hexagonal channel with a pore diameter of 12.8 Å. Most importantly, we found that all the incorporated —NH— polar groups in Zr‐adip are surrounded the triangular and hexagonal pore surfaces, and point into the hexagonal pores. Therefore, these immobilized Lewis basic N sites within Zr‐adip may not only enhance the pore hydrophilia but also serve as additional binding sites to moderately interact with water molecules to improve water uptake capacities at low relative pressure, thereby promoting the COP values for heating and cooling applications.

**Figure 2:**
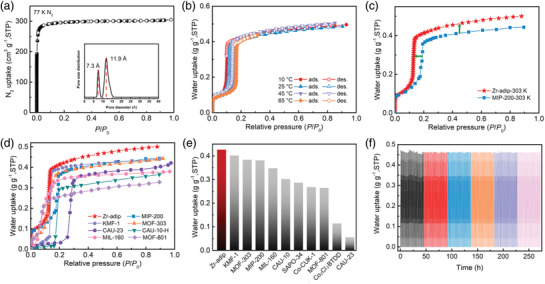
a) N_2_ adsorption isotherms of Zr‐adip at 77 K. Filled/empty symbols represent adsorption/desorption. Inset shows pore size distribution of Zr‐adip calculated based on DFT model. b) Water adsorption isotherms of Zr‐adip at different temperatures. c) Comparison of water adsorption isotherms for Zr‐adip with MIP‐200. d) Water adsorption isotherms of Zr‐adip and some top‐performing materials at 303 K, except CAU‐23 at 298 K. e) Comparison of the water uptake of Zr‐adip and other best‐performing MOFs at *P*/*P*_0_ = 0.25 and 303 K, except CAU‐23 and Co_2_Cl_2_BTDD at 298 K. f) Thermogravimetric analysis profile of multiple water adsorption‐desorption tests for Zr‐adip over 300 continuous cycles.

The permanent porosity of activated Zr‐adip was established by nitrogen (N_2_) adsorption isotherms at 77 K. Similar to the isoreticular MIP‐200, the as‐synthesized Zr‐adip sample was first treated with concentrated HCl to replace most of the terminal formate as the hydroxyl groups or water molecules before N_2_ adsorption measurements. Such HCl treatment leads to an obvious increase on the N_2_ uptake at 77 K for the activated Zr‐adip (Figure S5, Supporting Information), implying the success of the formate replacement. Such formate replacement was also confirmed by ^1^H NMR spectroscopy (Figure S6, Supporting Information), in which the hydrogen signal of formate groups from the sample with HCl treatment almost disappeared. As shown in **Figure** **2**a, Zr‐adip shows a significant type I sorption behavior without hysteresis, taking up a large amount of N_2_ (305.2 cm^3^ g^−1^) at 77 K and 1 bar. The Brunauer–Emmett–Teller surface area and pore volume of Zr‐adip were calculated to be 1214 m^2^ g^−1^ (Figure S5, Supporting Information) and 0.47 cm^3^ g^−1^, respectively. Both of the values are higher than those of the isoreticular MIP‐200 (1000 m^2^ g^−1^ and 0.40 cm^3^ g^−1^),^[^ ^69^ ^]^ mainly attributed to the incorporation of smaller —NH— spacer to replace —CH_2_— group. The pore size distribution, determined by the DFT model based on 77 K N_2_ adsorption, exhibits two types of pores with a diameter of 7.3 and 11.9 Å, respectively (Figure 2a), which is compatible with the results observed from the crystal structure.

### 2.2 Water Sorption Properties

Water sorption isotherms of the activated Zr‐adip were measured at 10, 25, 45, and 65 °C, respectively, as shown in Figure 2b and Figure S7, Supporting Information. Zr‐adip exhibits steep‐like adsorption isotherms with a fully reversible desorption branch, in which two adsorption steps exist at *P*/*P*_0_ < 0.05 and at *P*/*P*_0_ changing from 0.10 to 0.18 as the temperature rises. Such S‐shaped water adsorption isotherms of Zr‐adip at room temperature enable that most of the water uptake occurs at *P*/*P*_0_ < 0.25. As shown in Figure 2c, compared with the isoreticular MIP‐200, the step pressure of Zr‐adip (*P*/*P*_0_ = 0.12) is much lower than that of MIP‐200 (*P*/*P*_0_ = 0.18), indicating a stronger water affinity due to the incorporation of hydrophilic N sites into the framework. At 303 K and *P*/*P*_0_ = 0.25, Zr‐adip exhibits an extremely high water uptake of 0.43 g g^−1^, significantly larger than that of MIP‐200 (0.39 g g^−1^).^[^ ^69^ ^]^ Such enhanced water uptake is probably attributed to the synergistic effect of the higher pore hydrophilia and the improved pore volume of Zr‐adip. It is worth noting that this uptake of Zr‐adip is even among the highest reported so far at the same conditions (Figure 2d,e and Table S3, Supporting Information), far surpassing that of KMF‐1 (0.39 g g^−1^),^[^ ^70^ ^]^ MOF‐303 (0.38 g g^−1^),^[^ ^71^ ^]^ MIL‐160 (0.35 g g^−1^),^[^ ^46^ ^]^ CAU‐23 (0.06 g g^−1^),^[^ ^57^ ^]^ and Co_2_Cl_2_BTDD (0.11 g g^−1^).^[^ ^59^ ^]^ In contrast, we found that the water uptake of Zr‐adip at ultra‐low relative pressures (<0.05) is almost the same with that of MIP‐200 (Figure 2c), implying that the incorporated N sites are not the primary and strong adsorption sites in Zr‐adip. These observations on the improvement of water uptake at *P*/*P*_0_ = 0.25 while having no effect at *P*/*P*_0_ < 0.05, induced by the immobilization of Lewis basic N sites, are able to maximize the working capacity and thus the performance for heating and cooling applications.

The experimental isosteric heat of water adsorption (−*Δ*_ads_ *H*) of Zr‐adip was calculated by applying the Clausius–Clapeyron equation based on water adsorption isotherms collected at four different temperatures (Figure 2b). As shown in Figure S8, Supporting Information, a moderate −*Δ*_ads_ *H* of 46–51 kJ mol^−1^ was observed for Zr‐adip at the main stage of water uptake. We found that the −*Δ*_ads_ *H* value of Zr‐adip is slightly larger than MIP‐200 at relatively high water uptakes of above 0.15 g g^−1^,^[^ ^69^ ^]^ further confirming that the incorporated N sites are not the primary and strong adsorption sites in Zr‐adip. We note that this value is comparable to most of the best‐performing MOFs such as KMF‐1 (52–57 kJ mol^−1^)^[^ ^70^ ^]^ and CAU‐23 (48.2 kJ mol^−1^),^[^ ^57^ ^]^ but notably lower than that of SAPO‐34 (50–60 kJ mol^−1^),^[^ ^13^ ^]^ strongly suggesting that the water desorption can be easily fulfilled at low driving temperatures since it is only slightly larger than the evaporation enthalpy of water (40.7 kJ mol^−1^). These results are also well consistent with the observations on water adsorption isotherms that show no any hysteresis loop between adsorption and desorption branches.

Kinetic sorption behaviors of Zr‐adip are another important factor to be considered for the real applications because the water adsorption/desorption cycle time directly impacts the heat transfer efficiency. The water adsorption/desorption kinetic profiles of Zr‐adip were shown in Figure S9, Supporting Information, measured with the operating conditions between adsorption at 25 °C/20% RH and desorption at 65 °C/0% RH. It was found that Zr‐adip reaches an adsorption saturation within 100 min under 20% RH and 25 °C. Subsequently, the adsorbed water can be totally desorbed within 20 min at 65 °C, verifying its ability of the low‐temperature desorption. We further evaluated the cyclability of Zr‐adip, deduced from the above gravimetric water‐sorption cycles. As shown in Figure 2f, the experimental water‐sorption cycling tests indicated that the adsorption performance of Zr‐adip almost remains intact with a negligible weight loss in water uptake (0.46 ± 0.02 g g^−1^) over 300 consecutive cycles, suggesting a very high cycling durability of this material.

**Figure 3:**
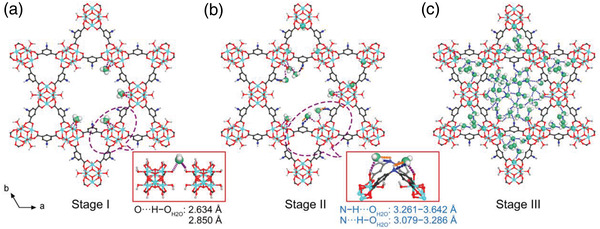
Simulation result of water adsorption process. a) The preferential adsorption site for water molecule in Zr‐adip at a very low pressure (*P*/*P*_0_ < 0.07). b) The primary water binding sites at the second adsorption stage (*P*/*P*_0_ ≈ 0.12). c) Densely packed water molecules in the hexagonal and triangular channels at saturation (*P*/*P*_0_ = 0.25) to form water clusters, obtained from theoretical simulations. Zr, blue; C, grey; O, red; H, white; O (in H_2_O), green.

To elucidate the origin of the ultrahigh water uptake observed for Zr‐adip, we performed grand canonical Monte Carlo simulations on Zr‐adip to investigate the interactions between the Zr‐adip framework and water molecules. The simulated water adsorption isotherms are shown in Figure S11, Supporting Information, in which both adsorbed tendency and adsorption amount are in good agreement with the experimental results. According to the water adsorption isotherms, the adsorption processes of Zr‐adip are composed of three typical stages. As shown in **Figure** **3**a, we found that the initial water adsorption sites are mainly located at the sides of two adjacent Zr_6_ SBUs along the *c* axis. Each water molecule interacts with the hydroxyl groups and water molecules coordinated to the metal centers through H···O_H2O_ hydrogen bonding with the distances of 2.634 and 2.850 Å, respectively. The calculated binding energy for this adsorption is very high with a value of 77.8 kJ mol^−1^, which should be associated with the first adsorption stage occurred at a very low relative pressure (*P*/*P*_0_ < 0.07). This initial adsorption site is well consistent with that observed in MIP‐200 in the literature,^[^ ^69^ ^]^ indicating that the incorporated N sites are not the primary and strong water binding sites and show no effect on water uptakes at this stage. When the population of adsorbed water molecules continues to grow until *P*/*P*_0_ = 0.12, some of water molecules gradually appear near the nitrogen atoms of the linkers in the hexagonal channels (Figure 3b). Each of these water molecules interacts not only with hydroxyl groups or coordinated water molecules from Zr_6_ SBUs through the formation of hydrogen bonding (2.634−2.850 Å), but also binds with nitrogen atoms to form N—H···O_H2O_ (H···O, 3.261−3.642 Å) and N···H—O_H2O_ interactions (N···H, 3.079−3.286 Å). In contrast, MIP‐200 only shows the H‐bonding interactions with hydroxyl groups and coordinated water molecules from Zr_6_ SBUs due to the absence of Lewis basic N sites. Evidently, the incorporated —NH— sites mainly contribute to the stronger binding affinity for water at this adsorption stage, thus leading to the improved water uptake for Zr‐adip at low relative pressure range. This is followed by a sudden increase in the water content at *P*/*P*_0_ = 0.12−0.20, in which more water molecules fill into the hexagonal and triangular channels through the formation of hydrogen bonds with the adsorbed molecules at the primary binding sites. As shown in Figure 3c, the pore‐filling mechanism finally involves a network of water molecules that interact with each other through strong hydrogen bonds. Overall, the introduction of Lewis basic N sites into Zr‐adip can serve as secondary adsorption sites to participate in the binding affinity increase above *P*/*P*_0_ = 0.07, thus resulting in the notably enhanced water uptake at *P*/*P*_0_ = 0.25.

### 2.3 AC/AHP Performance Assessments

The excellent water sorption properties of Zr‐adip inspired us to evaluate its achievable thermodynamic performance for AC and AHP applications at low driving temperatures (e.g., 70 °C). A full assessment and understanding of the thermodynamic cycle between an adsorbent and water as an adsorbate are shown in Figure S12, Supporting Information.^[^ ^6^ ^]^ They are calculated by thermodynamic models applied at different boundary temperature conditions for water evaporation (*T*_ev_), condensation (*T*_con_), adsorption (*T*_ads_), and desorption/regeneration (*T*_des_). Standard boundary conditions of these four temperatures used to screen the performances of water adsorbents have been already established, as depicted in Table S4, Supporting Information.^[^ ^6^, ^44^ ^]^ The COP for cooling and heating were thus evaluated on Zr‐adip under the standard operating conditions of refrigeration and heating based on the well‐established calculation methods.^[^ ^6^ ^]^

**Figure 4:**
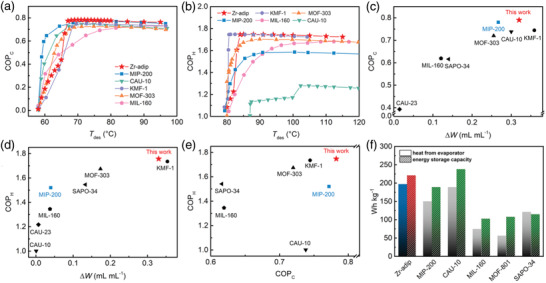
a) COP_C_ plots for AC conditions (*T*_ev_ = 5 °C, *T*_ads_ = 30 °C) as a function of desorption temperature (*T*_des_). b) COP_H_ plots for AHP conditions (*T*_ev_ = 15 °C, *T*_ads_ = 45 °C) as a function of *T*_des_. c) COP_C_ versus volumetric working capacity (Δ*W*) defined as the volume of liquid water per volume of dry adsorbent, examined under standard AC conditions (*T*_ev_ = 5 °C, *T*_ads_ = 30 °C, *T*_con_ = 30 °C, and *T*_des_ = 70 °C). d) COP_H_ versus volumetric working capacity (Δ*W*) defined as the volume of liquid water per volume of dry adsorbent, examined under standard AHP conditions (*T*_ev_ = 15 °C, *T*_ads_ = 45 °C, *T*_con_ = 45 °C, and *T*_des_ = 85 °C). e) Comparison of COP_C_ and COP_H_ values of Zr‐adip with the indicated best‐performing materials at the same conditions. f) Heat from evaporator and heat‐storage capacities for Zr‐adip and some promising adsorbents expressed in gravimetry scales. Boundary conditions: heats transferred from the evaporator in one cooling cycle at *T*_ev_ = 10 °C, *T*_ads_ = 30 °C, *T*_con_ = 30 °C, and *T*_des_ = 70 °C (blank bars) and heat‐storage capacity at *T*_ev_ = 10 °C, *T*_ads_ = 30 °C, *T*_con_ = 30 °C, and *T*_des_ = 70 °C (diagonal bars).

For AC applications, COP for cooling (COP_C_) is defined as the useful energy output (*Q*_ev_) that is withdrawn by the evaporator, divided by the energy source required as an input (*Q*_des_) for the regeneration of the adsorbent. The calculated COP_C_ curves for Zr‐adip as a function of desorption temperature (*T*_des_) are depicted in **Figure** **4**a under the given standard refrigeration conditions (i.e., *T*_ev_ = 5 °C and *T*_con_ = 30 °C), and compared with some best‐performing water adsorbents reported. With the *T*_des_ increased, the COP_C_ value of Zr‐adip rises gradually below 65 °C and then reaches maximum at 67 °C, indicating that Zr‐adip has the potential for ultralow temperature driven ACs. At a low driving temperature of 70 °C, Zr‐adip exhibits an extremely high COP_C_ value of 0.79. This value is slightly higher than that of the isoreticular MIP‐200 (0.78)^[^ ^69^ ^]^ and most of the benchmark MOFs such as KMF‐1 (0.75),^[^ ^70^ ^]^ MOF‐303 (0.72),^[^ ^71^ ^]^ and CAU‐23 (0.40).^[^ ^57^ ^]^ When *T*_des_ is higher than 70 °C, Zr‐adip shows a very high volumetric working capacity (Δ*W*) over 0.316 mL mL^−1^ for AC applications, absolutely higher than that of MIP‐200 (0.258 mL mL^−1^). This working capacity also surpasses most promising water adsorbents like SAPO‐34 (0.136 mL mL^−1^), CAU‐10 (0.297 mL mL^−1^), MOF‐303 (0.257 mL mL^−1^), and CAU‐23 (0.017 mL mL^−1^).^[^ ^70^ ^]^ If we set the COP_C_ and volumetric working capacity as concurrent objectives, Zr‐adip shows a rare combination of simultaneously high COP_C_ and working capacity, rendering it as one of the best water adsorbents for AC applications. In addition, Zr‐adip also keeps a very high value in terms of working capacity and COP_C_ in another cooling condition, as indicated in Figure S13, Supporting Information.

With respect to AHP applications, COP for heating (COP_H_) is defined as the sum of the two useful energy outputs during the adsorption process, divided by the energy input (*Q*_reg_) required for the adsorbent regeneration. Two energy outputs are the energy for condensation (*Q*_con_) and the energy for adsorption (*Q*_ads_). As shown in Figure 4b, Zr‐adip exhibits an exceptionally high COP_H_ value on heating at *T*_ev_ = 15 °C, *T*_con_ = 45 °C, and *T*_des_ = 85−100 °C. When setting *T*_des_ = 85 °C, Zr‐adip shows an exceptionally high COP_H_ value of 1.75, which is significantly higher than that of MIP‐200 (1.53)^[^ ^69^ ^]^ and comparable to the previously best KMF‐1 (1.74).^[^ ^70^ ^]^ Further, Zr‐adip shows a high working capacity of 0.324 mL mL^−1^ for AHP applications, six times larger than MIP‐200 (0.048 mL mL^−1^).^[^ ^69^ ^]^ As shown in Figure 4d, Zr‐adip achieves a notable advancement with both high COP_H_ and working capacity for AHP applications. Figure 4e further presents a comprehensive comparison of Zr‐adip with the top‐performing adsorbents in light of COP_C_ and COP_H_ as concurrent objectives. Among these indicated water adsorbents, Zr‐adip exhibits both the highest COP_C_ and COP_H_ values (0.79 and 1.75), surpassing the performance of the previous benchmark KFM‐1 (0.75 and 1.74),^[^ ^70^ ^]^ MIP‐200 (0.78 and 1.53), and MOF‐303 (0.72 and 1.68).^[^ ^69^ ^]^ Taken together, this material is placed among the best existing materials for both AC and AHP applications.

Considering the excellent cooling and heating performance of Zr‐adip, the gravimetric heat values from the evaporator and heat‐storage capacities were also estimated and compared with other promising adsorbents under specific working conditions. As shown in Figure 4f, Zr‐adip exhibits the high values of 197.3 and 221.2 Wh kg^−1^ for both the evaporative and heat storage capacity in a single refrigeration cycle (at *T*_ev_ = 10 °C, *T*_ads_ = 30 °C, and *T*_des_ = 70 °C), which are comparable to CAU‐10‐H (189 and 238 Wh kg^−1^), but far surpass MIP‐200 (150.2 and 189 Wh kg^−1^) and the commercial SAPO‐34 (121.4 and 115 Wh kg^−1^). Moreover, the capacities can be further increased to 222.4 and 250.2 Wh kg^−1^ at higher regeneration temperatures (*T*_des_ = 80 °C), as shown in Table S5 and Figures S16–S18, Supporting Information.

### 2.4 Chemical Stability

**Figure 5:**
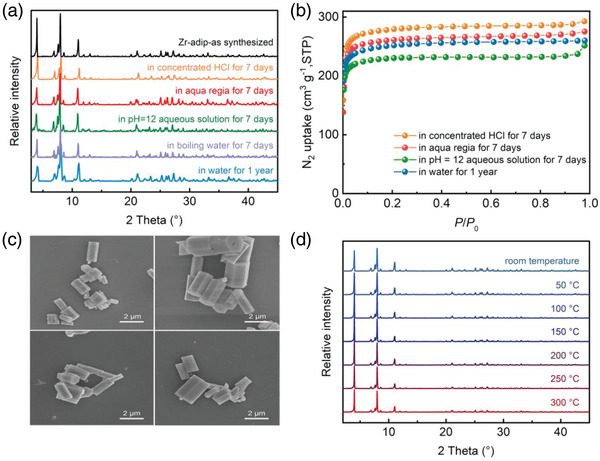
a) PXRD patterns of Zr‐adip samples after treatment with different conditions. b) N_2_ adsorption isotherms of Zr‐adip at 77 K after the sample soaking in water for 1 year or treatment under different conditions, indicating its highly chemical stability. c) SEM images of Zr‐adip after the treatments in boiling water (top left), concentrated HCl (12 m) (top right), aqua regia (bottom left), and pH = 12 aqueous solution (bottom right) for 2 days. d) Variable‐temperature PXRD patterns for Zr‐adip.

Actual AHP/ACs operating cycles would be performed under harsh and complicate conditions in industrial applications, which require water adsorbents to have extremely high and long‐term water and chemical stability. We first investigated the long‐term water stability of Zr‐adip by soaking the as‐synthesized sample into water for 1 year, monitored by PXRD and N_2_ adsorption measurements. As shown in **Figure** **5**a,b, Zr‐adip maintains its crystal structure in water as evidenced by the almost identical PXRD patterns and N_2_ adsorption. To further confirm the water stability of Zr‐adip, the sample was soaked in water for 7 days and then the supernatant was collected for the inductively coupled plasma mass spectrometry (ICP‐MS) measurements. The concentration of Zr^4+^ ions in analyzed sample was found to be very low of 1.169 ppm, indicating that the Zr‐adip framework does not decompose during water sorption. Further, we also investigated the chemical stability of Zr‐adip toward different acid–base solutions. We exposed Zr‐adip samples to different chemical environments for 7 days, including concentrated HCl, aqua regia, and a buffer of pH 12. The Scanning electron microscope (SEM) images of the crystals of Zr‐adip show that there is no apparent change in their morphology after the treatment with various pH solutions (Figure 5c). As shown in Figure 5a, the framework of Zr‐adip is stable in these extremely harsh conditions for 7 days, and no loss in crystallinity and no phase change were observed by the PXRD. Moreover, N_2_ adsorption isotherms at 77 K after treatment are very close to those of as‐synthesized materials, further confirming its ultrahigh chemical stability. In addition, the variable temperature PXRD patterns indicate that Zr‐adip is thermally stable up to 300 °C (Figure 5d). Therefore, this material shows one of the best chemical stability performances among all the reported MOFs, even comparable to some of the state‐of‐the‐art MOFs and COFs.

## 3 Conclusion

In summary, we have demonstrated that the immobilization of Lewis basic nitrogen sites into MOFs can boost water sorption performance for highly efficient adsorption‐driven cooling and heating. Our foregoing results revealed that the incorporated N sites within Zr‐adip can enhance pore hydrophilicity and serve as secondary adsorption sites to moderately interact with water molecule, while maintaining its high surface area and low regeneration temperature, as supported by the sorption studies and theoretical calculations. Zr‐adip thus exhibits typically S‐shaped adsorption isotherms with an unprecedented water uptake of 0.43 g g^−1^ at 303 K and *P*/*P*_0_ = 0.25, which is notably higher than MIP‐200 (0.39 g g^−1^) and other benchmark MOFs like KMF‐1 (0.39 g g^−1^) and MOF‐303 (0.38 g g^−1^). As a consequence, this material displays a rare combination of both exceptionally high COP_C_ (0.79) and COP_H_ (1.75) values with high volumetric working capacities at a low driving temperature of 70 °C, exceeding most of the best‐performing materials reported so far. The combined features of this material including exceptional water uptake capacity, benchmark COP values at low driving temperature, ultrahigh stability, and excellent recyclability, make it one of the most promising adsorbents for AHP and AC applications. These results revealed in this work may provide an alternative strategy to design and functionalize porous MOFs with Lewis basic N sites to target high‐performance AC and AHP.

## 4 Experimental Section

### Materials and Methods

All starting chemicals and solvents were purchased from commercial companies and used without further purification. 3,3′,5,5′‐tetracarboxydiphenylmethane (H_4_mdip) (CAS: 10397‐52‐1) was purchased from Energy Chemical, and ZrCl_4_ (CAS: 10026‐11‐6) was purchased from Aladdin. 5,5′‐azanediyldiisophthalic acid (H_4_adip) was prepared according to the previous literatures, more details can be found in the Supporting Information. ^1^H spectra was recorded on Bruker AVANCE III spectrometers (300 and 500 MHz). PXRD patterns were collected by a BRUKER D8 ADVANCE diffractometer employing Cu‐K*α* (*λ* = 1.542 Å) radiation operated at 30 kV and 15 mA, scanning over the range 2–45° (2*θ*) at a rate of 5° min^−1^ under ambient conditions. ICP‐MS was performed on a Thermo Scientific XSERIES 2 ICP‐MS system. SEM patterns were observed by Hitachi S‐4800 field emission SEM. Thermogravimetric analyses were performed on a TA SDT 650 thermal analyzer from 30 to 800 °C under nitrogen atmosphere at a heating rate of 10 °C min^−1^ rate. All of the computations in this work were based on the previous literature methodologies, which could be found in Supporting Information.

### Synthesis of Zr‐adip

5,5′‐azanediyldiisophthalic acid (42.5 mg, 0.12 mmol) and ZrCl_4_ (51 mg, 0.2 mmol) were dissolved in acetic anhydride (3.5 mL) by sonicating for 15 min. Then, 2.5 mL of formic acid, two drops of acetic acid by using a dropper (1 mL), and 20 µL HCl were added and the mixture was sonicated for an additional 10 min. For safety, the sonicating temperature should not be too high due to the low boiling point of formic acid. The reactants were then added to a Teflon‐lined autoclave and heated at 120 °C for 48 h. After being filtered and washed with DMF and methanol at least three times, Zr‐adip was obtained as white powder sample.

### Gas Sorption Measurements

The fresh sample (50–100 mg) should be soaked in concentrated HCl solution at 80 °C for 24 h, and then washed with DMF and solvent‐exchanged with dry ethanol at least eight times within 3 days before the sorption test to thoroughly eliminate the guest solvent molecules in the channel. N_2_ sorption isotherms were measured by the Micromeritics ASAP 2460 surface area analyzer and the measurement was maintained at 77 K with liquid nitrogen. Before measurement, the solvent‐exchanged sample was evacuated at room temperature for 12 h and further at 363 K for 12 h until the outgas rate was 4 *μ*mHg min^−1^. Volumetric water sorption isotherms were measured by the BELSORP‐max instrument. All water analyses were performed using water baths held at constant temperature with a recirculating chiller. Before measurement, the solvent‐exchanged sample was evacuated at room temperature for 12 h and further at 373 K for 12 h under high vacuum (<0.2 Pa).

## Conflict of Interest

The authors declare no conflict of interest.

## Supporting information

Supporting Information

## Data Availability

The data that support the findings of this study are available from the corresponding author upon reasonable request.
