# Supplementary material for: Immobilization of Lewis Basic Nitrogen Sites into a Chemically Stable Metal–Organic Framework for Benchmark Water‐Sorption‐Driven Heat Allocations
Source: Adv Sci (Weinh). 2022 Feb 11;9(11):2105556. doi: 10.1002/advs.202105556 (PMC9009103; doi:10.1002/advs.202105556)
Supplement: Supplementary file 1 — Supporting Information [file ADVS-9-2105556-s001.pdf]

ADVANCED SCIENCE
Open Access

Supporting Information

for *Adv. Sci.*, DOI 10.1002/advs.202105556

Immobilization of Lewis Basic Nitrogen Sites into a Chemically Stable Metal–Organic Framework for Benchmark Water-Sorption-Driven Heat Allocations

Bin Li, Feng-Fan Lu, Xiao-Wen Gu, Kai Shao, Enyu Wu and Guodong Qian**

ADVANCED SCIENCE
Open Access

Supporting Information

for *Adv. Sci.,* DOI: 10.1002/advs.202105556

Immobilization of Lewis Basic Nitrogen Sites into a Chemically Stable Metal–Organic Framework for Benchmark Water-Sorption-Driven Heat Allocations

*Bin Li, * Feng-Fan Lu, Xiao-Wen Gu, Kai Shao, Enyu Wu, and Guodong Qian**

Supporting Information

Immobilization of Lewis Basic Nitrogen Sites into a Chemically Stable Metal–Organic Framework for Benchmark Water-Sorption-Driven Heat Allocations

Bin Li, [+] Feng-Fan Lu,[+] Xiao-Wen Gu, Kai Shao, Enyu Wu, and Guodong Qian**

Prof. B. Li, F.-F. Lu, X.-W. Gu, K. Shao, E. Wu, Prof. G. Qian

State Key Laboratory of Silicon Materials, School of Materials Science and Engineering, Zhejiang University, Hangzhou 310027, China

E-mail: bin.li@zju.edu.cn; gdqian@zju.edu.cn

[+] These authors contributed equally to this work.

Experimental procedure

Synthesis of 5,5'-azanediyldiisophthalic acid

H_4adip was synthesized according to previous literature (Scheme S1).[1] Dimethyl 5-(((trifluoromethyl)sulfonyl)oxy)isophthalate (1.71 g, 5 mmol), dimethyl 5-aminoisophthalate (1.54 g, 6 mmol), Cs_2CO_3 (2.28 g, 7 mmol), BINAP (0.248 g, 0.4 mmol) and $Pd(OAC)_2$ (0.056 g, 0.25 mmol) were dissolved in dry toluene. The mixture was stirred at 120 °C for two days, then cooled to room temperature. The reaction mixture was filtered through celite, and the solid was purified by flash chromatography over silica (20% EtOAc in hexanes) to afford Me_4adip as a white solid. Yield: 33%. Me_4adip (2.01 g, 5 mmol) was suspended in 100 mL 6M NaOH/THF/MeOH (1:1:1 v/v/v). The mixture was stirred under reflux for 24 h. After that, THF and MeOH were removed under reduced pressure and dilute HCl was then added to the remaining aqueous solution to acidify pH = 2. The precipitate was collected by filtration, washed with water for several times, and dried to afford white powder. Yield: 95%. ^{1}H NMR (300 MHz, DMSO-d6) δ 13.24 (s, 4H), 9.04 (s, 1H), 8.00 (t, J = 1.5 Hz, 2H), 7.87 (d, J = 1.5 Hz, 4H).

Synchrotron X-ray diffraction experiments

Synchrotron X-ray diffraction (SXRD) patterns were collected on the BL14B1 employing microfocused X-rays (**λ** = 0.6887 Å**)** at the Shanghai Synchrotron Radiation Facility (SSRF). The quartz capillary with the diameter of 1 mm was used to place the test sample, and was constantly revolved during the whole measurement. The two-dimensional XRD signal was obtained by a Mythen 1K linear detector. Through indexing of the SXRD data, a hexagonal *P*6/mmm space group was first identified for the Zr-adip crystal. We thus modeled its structure using the same framework connection as MIP-200. Based on the structure model, Rietveld refinement was performed on the SXRD data, and we obtained the unit cell parameters of $a = b = 25.5265$ Å and $c = 11.5812$ Å with agreement factors of $R_p = 0.0384$ and $R_{wp} = 0.0680$ for Zr-adip (Figure S4, Supporting Information), strongly supporting its validity. Detailed structure information of Zr-adip is provided in Table S1 and S2 (Supporting

Information).

Grand Canonical Monte Carlo simulations

The binding sites of water molecules in the structure of Zr-adip were identified using Grand Canonical Monte Carlo (GCMC) simulations. The GCMC simulations were carried out using the Sorption program in BIOVIA Material studio 8.0. In this work, the crystal structure of Zr-adip was chosen for related simulations without further geometry optimization. The interaction energy between water molecules and framework were computed through the Coulomb and Lennard-Jones 12−6 (LJ) potentials. A cutoff radius of 12.5 Å was used to handle the nonbonding interactions, and the Ewald & Group summation method was applied to calculate the long-range electrostatic interactions. The MOF frameworks were treated as rigid structures by fixing atoms at their crystallographic positions, and the simulation box was made of 12 (2 × 2 × 3) unit cells of Zr-adip. A mixed set of UFF and DREIDING force field parameters were adopted to describe the LJ parameters for the atoms in Zr-adip framework. Partial charges for atoms of guest-free Zr-adip were derived from QEq method and QEq_neutral1.0 parameter. Water molecules were described by the TIP4P/2005 potential model. The details of the force field parameters are summarized in Tables S6 and S7. The Lorentz-Berthelot combining rules were employed to calculate cross interactions. The loading steps and the equilibration steps were 1×10^7, the production steps were 1×10^7.

Kinetic measurement and cyclability test of water adsorption/desorption

A thermal gravimetric analyzer (TGA, SDT 650, TA Instruments) was used to measure the water adsorption/desorption kinetics in conjunction with a humidity generator. The schematic diagram of this kinetic measurement instrument was shown in Scheme 2. The humidification system is made up of two N_2 purge gases: one runs inside liquid water transporting water vapor (100% RH), and the other is dry N_2, and then blended in a humidifier-mixer. A highly accurate humidity sensor was used to determine the humidity of the mixture gas before it passed through the thermogravimetric balance. The humidity of the mixture gas can be

changed between 0% and 100% RH by adjusting the flux of these two purge gases. Adsorption patterns were obtained at 25 °C in humid nitrogen with 20% RH or 40% RH for kinetic measurements, and desorption profiles were taken at 65 °C or 85 °C in dry nitrogen with 0% RH. The experimental water-sorption cycling tests were carried out at 30 °C in humid nitrogen with 40% RH for the weight-gain profiles, and collected at 100 °C with 0% RH for the weight-loss profiles.

Calculation of the characteristic curves of Zr-adip.

The Dubinin model is used to represent the adsorption equilibrium, or the loading of the adsorbent *W* as a function of temperature *T* and pressure *P*. The volume occupied by the adsorbed phase in terms of the amount adsorbed is defined as:

$$W = \frac{\mathrm{q}(P,T)}{\rho_{\mathrm{liq}}^{\mathrm{wf}}(T)} \quad (1)$$

The characteristic curve is used to make the loading dependent on only one free variable (“energy”) rather than two (*P*, *T*). The energy parameter employed is the adsorption potential (*A*), which is the inverse sign of the Gibbs free energy of adsorption:

$$A = -\Delta G = RT\ \ln\left(\frac{P_0(T)}{P}\right) \quad (2)$$

P_0 represents the saturation vapor pressure of the working fluid at the measurement temperature, *P* represents the pressure at each loading value, *R* represents the ideal gas constant, and *T* represents the analysis temperature. Isotherms measured at different temperatures should collapse onto a single characteristic curve. Water isotherms of Zr-adip were measured at 283 K, 298 K, 318 K and 338 K. (Figure 2b and Figure S7, Supporting Information). When each of these isotherms is used to calculate the characteristic curve, they all collapse into a single characteristic curve (Figure S14 and S15, Supporting Information), proving the validity of the characteristic curve concept.

Calculation of the coefficient of performance for Zr-adip.

An express method relies on De Lange et al. is used to calculate the thermodynamics of adsorption chiller and heat pump cycles.[3] From a thermodynamic standpoint, the coefficient

of performance (COP) is used to represent the energy efficiency of the heat pump cycle. The COP, which is a ratio of useful heating or cooling energy output to effort required, can be calculated using the energy analysis. Knowledge of the enthalpy of adsorption is essential for determining the COP of a working pair. The Clausius–Clapeyron equation is used to calculate the isosteric enthalpy of adsorption:[4]

$$\Delta_{\mathrm{ads}}H_W = R\left(\frac{\partial \ln P}{\partial(\frac{1}{T})}\right)w \quad (3)$$

where $\Delta_{\mathrm{ads}}H_{\mathrm{W}}$, R, P, T, and W represent the isosteric enthalpy of adsorption (kJ mol^{-1}), universal gas constant (kJ mol^{-1} K^{-1}), pressure (bar), temperature (K), and volume liquid adsorbed (mL(liq) g^{-1}), respectively. The heating and cooling coefficient of performance is stated as:

$$\mathrm{COP_C} = \frac{Q_{\mathrm{ev}}}{Q_{\mathrm{reg}}} \quad (4)$$

$$\mathrm{COP_H} = \frac{-(Q_{\mathrm{con}} + Q_{\mathrm{ads}})}{Q_{\mathrm{reg}}} \quad (5)$$

The COP can be estimated by multiplying the energy required for each stage as shown in Figure S12 (Supporting Information). The energy released for III-IV and IV-I is equal to the adsorption energy (Q_{ads}), whereas the energy required for I-II and II-III is the energy necessary for desorption ($Q_{\mathrm{reg/des}}$). In general, the adsorption temperature T_{ads} (T_1) is defined to be the same as the condenser temperature (T_{con}). T_2 is determined for a specific working pair by selecting the condenser's temperature and pressure based on its relationship with T_{con} via maximal water uptake (W_{max}). T_4 is related to T_{des} by the minimum water uptake (W_{min}), and T_4 is calculated when the evaporator temperature is decided. At the isobaric adsorption step (IV-I) of the adsorption ideal cooling thermodynamic cycle, the adsorbent is fully saturated up to maximum water absorption (W_{max}) and requires water regeneration or desorption. Pressure must be increased from evaporation pressure (P_{ev}) to condensation pressure (P_{con}) before water is released to the condenser, equivalent to the isosteric heating process (I-II). At the isobaric desorption step (II-III), adsorbent heating is continued and water is allowed to desorb up to minimum water uptake (W_{min}) and no further pressure increase occurs until desorption temperature (T_{des}) is reached. The isosteric cooling step (III-IV) is used to reduce the pressure at minimum water uptake (W_{min}). The pressure must be decreased from P_{con} to P_{ev} by cooling the adsorbent vessel from T_{des} to T_4, then reconnecting the condenser and

evaporator. Water is permitted to adsorb in the isobaric adsorption process, but no further pressure drop occurs. The evaporator continues to cool at this point. The energy required for each stage can be described as:

Isosteric heating (I-II):

$$Q_{\text{I-II}} = \int_{T_{\text{ads}}}^{T_2} c_P^{\text{eff}}(T)\,dT + \int_{T_{\text{ads}}}^{T_2} \rho_{\text{liq}}^{\text{wf}}\, W_{\max}\, c_P^{\text{wf}}(T)\,dT \quad (6)$$

Isobaric desorption (II-III):

$$Q_{\text{II-III}} = \int_{T_2}^{T_{\text{des}}} c_P^{\text{eff}}(T)\,dT + \int_{T_2}^{T_{\text{des}}} \rho_{\text{liq}}^{\text{wf}}\, \frac{W_{\max}+W_{\min}}{2}\, c_P^{\text{wf}}(T)\,dT - Q_{\text{sorption}} \quad (7)$$

Isosteric cooling (III-IV):

$$Q_{\text{III-IV}} = \int_{T_4}^{T_{\text{des}}} c_P^{\text{eff}}(T)\,dT + \int_{T_{\text{des}}}^{T_4} \rho_{\text{liq}}^{\text{wf}}\, W_{\max}\, c_P^{\text{wf}}(T)\,dT \quad (8)$$

Isobaric adsorption (IV-I):

$$Q_{\text{IV-I}} = \int_{T_{\text{ads}}}^{T_4} c_P^{\text{eff}}(T)\,dT + \int_{T_{\text{ads}}}^{T_4} \rho_{\text{liq}}^{\text{wf}}\, \frac{W_{\max}+W_{\min}}{2}\, c_P^{\text{wf}}(T)\,dT + Q_{\text{sorption}} \quad (9)$$

The effective heat capacity (c_P^{eff}) is considered to be the same as the heat capacity of sorbent (c_P^{sorbent}). Isosteric heating (I-II) and isobaric desorption (II-III) combine to provide the entire energy required for adsorbent regeneration.

$$Q_{\text{reg}} = Q_{\text{I-II}} + Q_{\text{II-III}} \quad (10)$$

Isosteric cooling (III-IV) and isobaric desorption (IV-I) combine to produce energy during the adsorption period of the heat pump cycle.

$$Q_{\text{ads}} = Q_{\text{III-IV}} + Q_{\text{IV-I}} \quad (11)$$

The energy released during adsorption of the working fluid (Q_{sorption}) can be written as

$$Q_{\text{sorption}} = \frac{1}{M_W} \int_{W_{\min}}^{W_{\max}} \rho_{\text{liq}}^{\text{wf}}\, \Delta_{\text{ads}}H(W)\,dW \quad (12)$$

The energy absorbed in the evaporator and released by the condenser can be calculated using the concept of enthalpy of evaporation, respectively.

$$Q_{\text{ev}} = -\frac{\Delta_{\text{vap}}H(T_{\text{ev}})\, \rho_{\text{liq}}^{\text{wf}}\, m_{\text{sorbent}}\, \Delta W}{M_W} \quad (13)$$

$$Q_{\text{con}} = \frac{\Delta_{\text{vap}}H(T_{\text{con}})\, \rho_{\text{liq}}^{\text{wf}}\, m_{\text{sorbent}}\, \Delta W}{M_W} \quad (14)$$

Chemical and thermal stability tests.

To investigate the chemical stability of Zr-adip, the as-synthesized samples were immersed in concentrated HCl, aqua regia, NaOH (pH = 12) solutions and boiling water for 7 days, respectively. Following that, each sample was filtered and washed in anhydrous ethanol before being analyzed using PXRD measurements to see if it kept structural integrity. Furthermore, the 77 K N_2 adsorption isotherms were measured to confirm the chemical stability of Zr-adip. Prior to gas sorption measurements, each sample was solvent-exchanged with dry ethanol at least 8 times within three days to remove the hardly volatile H_2O molecules in the pores completely, and then was evacuated at room temperature for 12 h and further at 373 K for 12 h. The thermal stability of Zr-adip was studied by thermogravimetric analysis and various temperature PXRD patterns. The sample for temperature PXRD patterns was treated for 12 h at 50 °C, 100 °C, 150 °C, 200 °C, 250 °C, and 300 °C, and then characterized by PXRD patterns. Meanwhile, the sample treated with 300 °C was also characterized by N_2 sorption measurements at 77 K.

List of symbols

Latin letters

Q energy, kJ mol^{-1}

m mass, g

W volume of liquid adsorbed, mL(liq) g^{-1}

q as a weight-ratio of the amount adsorbed, g g^{-1}

A adsorption potential, kJ mol^{-1}

ΔW working capacity, mL(liq) g^{-1}

R ideal gas constant, J K^{-1} mol^{-1}

T temperature, K

P pressure, kpa

P/P_0 relative pressure, also called as relative humidity for water adsorption

P_0 saturation pressure

c_p heat capacity, J g^{-1} K^{-1}/ (J mol^{-1} K^{-1})

M_w molar mass, g mol^{-1}

Greek symbols

$\Delta_{ads}H$ enthalpy of adsorption, kJ mol^{-1}

$\Delta_{vap}H$ enthalpy of evaporation, kJ mol^{-1}

ρ liquid density of the adsorbate, g cm^{-3}

Subscripts

ev evaporation

con condensation

ads adsorption

reg regeneration

des desorption

liq liquid

Superscripts

eff effective

wf working fluid

Abbreviation

refrig refrigeration

Table S1. Experimental data on Rietveld refinement of Zr-adip.

Unit cell parameters	Zr-adip
Formula	$C_{36}H_{22}N_2O_{32}Zr_6$
Formula weight	1541.89
Crystal system	Hexagonal
Space group	*P*6/mmm
a (Å)	25.5265
b (Å)	25.5265
c (Å)	11.5812
α (°)	90
β (°)	90
γ (°)	120
V (Å^3)	6535.36
Z	3
D_{calcd} (g cm^{-3})	1.17
R_p, %	3.846
Rw_p, %	6.802

Table S2. List of atomic coordinates for the modeled structure of Zr-adip.

Atoms	x	y	z	s.o.f
O1	0.528100	0.366600	0.885800	1.00
O2	0.449000	0.359800	0.785500	1.00
C3	0.482500	0.341900	0.816200	1.00
C4	0.469300	0.282200	0.757600	1.00
C5	0.433600	0.261900	0.660900	1.00
H6	0.422300	0.288510	0.625660	1.00
Zr145	0.554353	0.445647	0.000000	1.00
Zr151	0.429897	0.429897	0.845083	1.00
O152	0.344500	0.344500	0.855500	1.00
O153	0.434200	0.434200	0.654500	1.00
O187	0.402800	0.461200	0.000000	1.00
O199	0.475200	0.524800	0.846900	1.00
C211	0.490000	0.245000	0.803000	1.00
H212	0.518210	0.259100	0.865600	1.00
C213	0.412500	0.206250	0.610000	1.00
N247	0.375000	0.187500	0.500000	1.00
H253	0.331450	0.165730	0.500000	1.00

Table S3. Comparison of the water-adsorption properties of Zr-adip with other benchmark materials.

Materials	Ligand	Pore structure	BET (m^2 g^{-1})	Pore volume[a] (cm^3 g^{-1})	α[b]	Water uptake[c] (g g^{-1})	Ref.
Zr-adip	H_4adip	1-D	1214	0.47	0.13	0.43	This work
MIP-200	H_4mdip	1-D	1000	0.40	0.18	0.39	[2]
CAU-10-H	BDC	1-D	635	0.25	0.18	0.30	[5]
Co-CUK-1	2,4-PrDC	1-D	510	0.26	0.12	0.27	[6]
MOF-303	3,5-PzDC	1-D	1119	0.54	0.15	0.38	[7]
KMF-1	2,5-PyDC	1-D	1130	0.47	0.14	0.39	[8]
CAU-23	2,5-TDC	1-D	1250	0.49	0.27	0.06	[9,10]
Co_2Cl_2BTDD	BTDD	1-D	1912	NA	0.29	0.11	[11]
MOF-801	FA	3-D	990	0.45	0.09	0.27	[12]
MIL-160	2,5-FDC	1-D, flexible	1070	0.40	0.08	0.35	[13]

[a] Pore volume is calculated from N_2 adsorption isotherm (at 77 K). Reported values are used where possible; otherwise these are estimated from N_2 isotherms.

[b] α is the relative humidity (RH) at which half of the total uptake at 95% RH is reached, measured at 303 K, unless CAU-23 and Co_2Cl_2BTDD were measured at 298 K.

[c] Water uptake value is taken at 25% RH, measured at 303 K, unless CAU-23 and Co_2Cl_2BTDD were measured at 298 K.

Table S4. Boundary temperature conditions in adsorption-driven heat allocations for the different applications concerned.[3]

	heat pump	refirg-I	refirg-II	ice making[a]
T_{ev}/K	288	283	278	268
T_{con}/K	318	303	303	298

[a] Because water freezes under zero degrees Celsius, it cannot be used as an ice-making working fluid.

Table S5. Comparison of the materials properties of Zr-adip and energy storage capacities with other benchmark materials.

Materials	Crystal density[a] (cm^3 g^{-1})	$-\Delta_{ads}H$[b] (kJ mol^{-1})	Water capacity[c]		Heat from evaporator[d] (Wh kg^{-1})	Energy storage capacity[e] (Wh kg^{-1})	Ref.
			(g g^{-1})	(mL mL^{-1})			
Zr-adip	1.17	50.6	0.269 (0.266)	0.324 (0.316)	197.3 (222.4)	221.2 (250.2)	This work
MIP-200	1.16	49.9	0.040	0.048	150.2	189	[2]
CAU-10-H	1.15	53.5	0.0027	0.003	189	238	[2,5,14]
MOF-801	1.59	58.4	0.017	0.028	56.4	108	[2,12]
MIL-160	1.15	52	0.086	0.092	74.7	103	[2,13]
SAPO-34	1.43	50-60	0.103	0.148	121.4	115	[2,15]

[a] Crystal density is calculated from crystal structure data.

[b] Heat of adsorption (isosteric or average of isosteric heat) is calculated from Clausius–Clapeyron equation.

[c] Working capacity deduced from water-sorption cycles for AHP conditions: T_{ev} = 15 °C, T_{ads} = 45 °C, and T_{con} = 45 °C at T_{des} = 85 °C (for AC conditions: T_{ev} = 5 °C, T_{ads} = 30 °C, and T_{con} = 30 °C at T_{des} = 70 °C for values in the parenthesis)

[d] Heat transferred from the evaporator in one refrigeration cycle at T_{ev} = 10 °C, T_{ads} = 30 °C and T_{des} = 70 °C (T_{des} = 80 °C for values in the parenthesis)

[e] Energy storage capacity per unit weight of adsorbent at T_{ev} = 10 °C, T_{ads} = 30 °C and T_{des} = 70 °C (T_{des} = 80 °C for values in the parenthesis).

Table S6. The Lennard-Jones parameters used for the framework atoms of Zr-adip.

MOFs	Atom type	σ (Å)	ε (K)
Zr-adip	C	3.55	73.82
		3.62	99.83
	H	2.85	7.65
		2.85	0.05
	O	3.03	48.19
	N	3.26	38.97
	Zr	2.78	34.72

Table S7. Partial charges and Lennard-Jones parameters of water molecules taken from TIP4P/2005 model.

Atom type	σ (Å)	ε (K)	q (e)
O_e	3.1589	93.200	0.0000
H_e	0.0000	0.0000	0.5564
M_e	0.0000	0.0000	-1.1128

Scheme 1. The synthesis process of H_4adip.

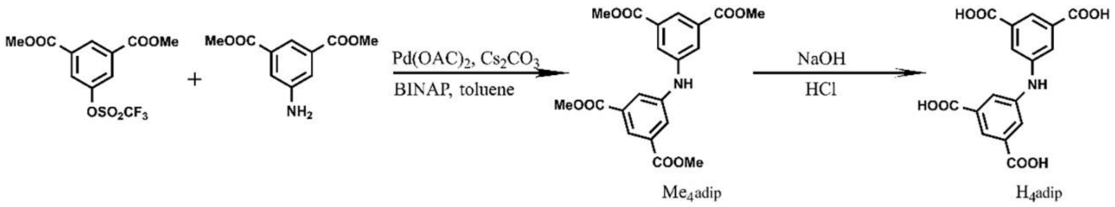

Scheme 2. Schematic diagram of the device for measuring the kinetics of water adsorption and desorption.

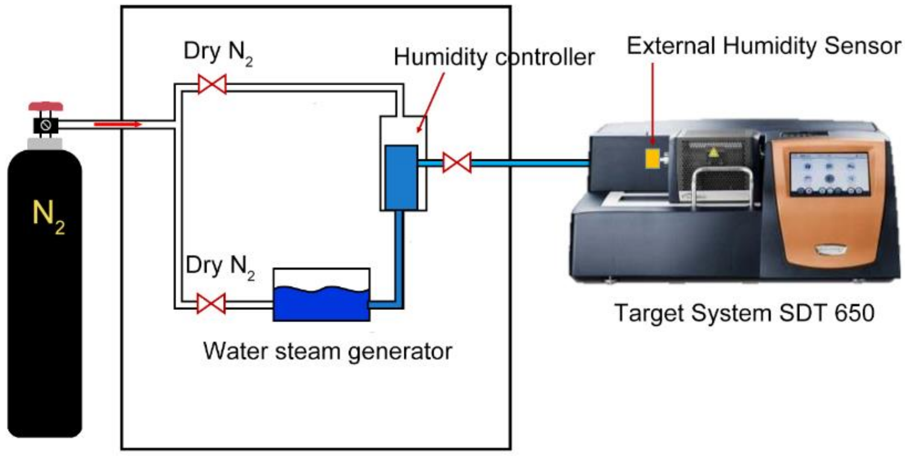

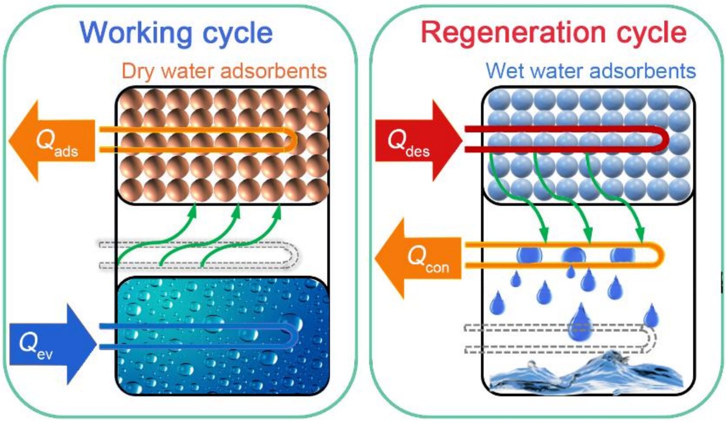

Figure S1. Principle of operation of an adsorption-driven heat cycle with the working stage (left) and the regeneration stage (right) Reproduced with permission.[16] Copyright 2013, Royal Society of Chemistry.

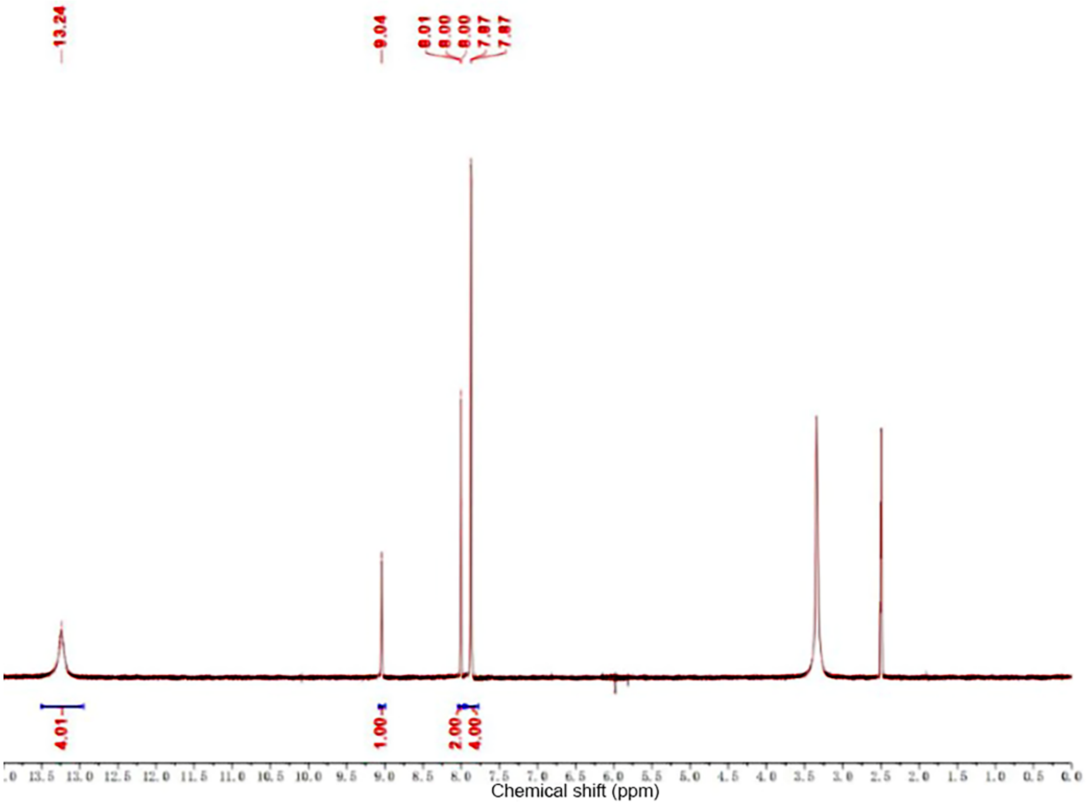

Figure S2. ^{1}H (DMSO-d_6, 300 MHz) spectra of H_4adip.

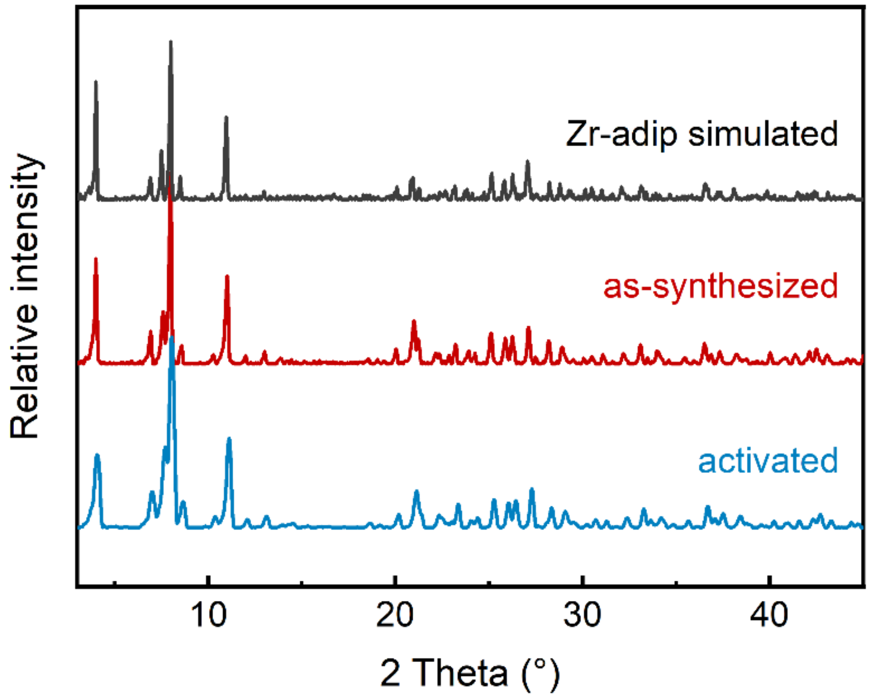

Figure S3. PXRD patterns of as-synthesized (red) and activated Zr-adip (blue) compared with the simulated XRD patterns from the structures of Zr-adip (black).

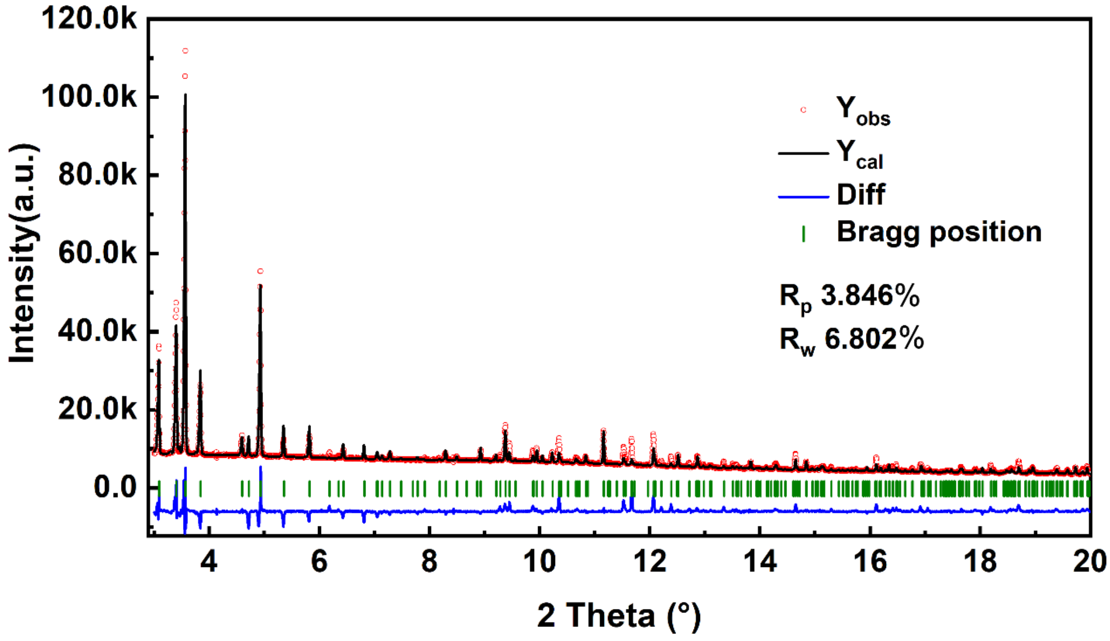

Figure S4. Rietveld refinement of the Zr-adip. The experimental data are shown in red, calculation in black, and difference in blue below. Allowed Bragg reflections are indicated as green ticks below. Goodness of fit data: R_p = 0.03846, R_{wp} = 0.06802.

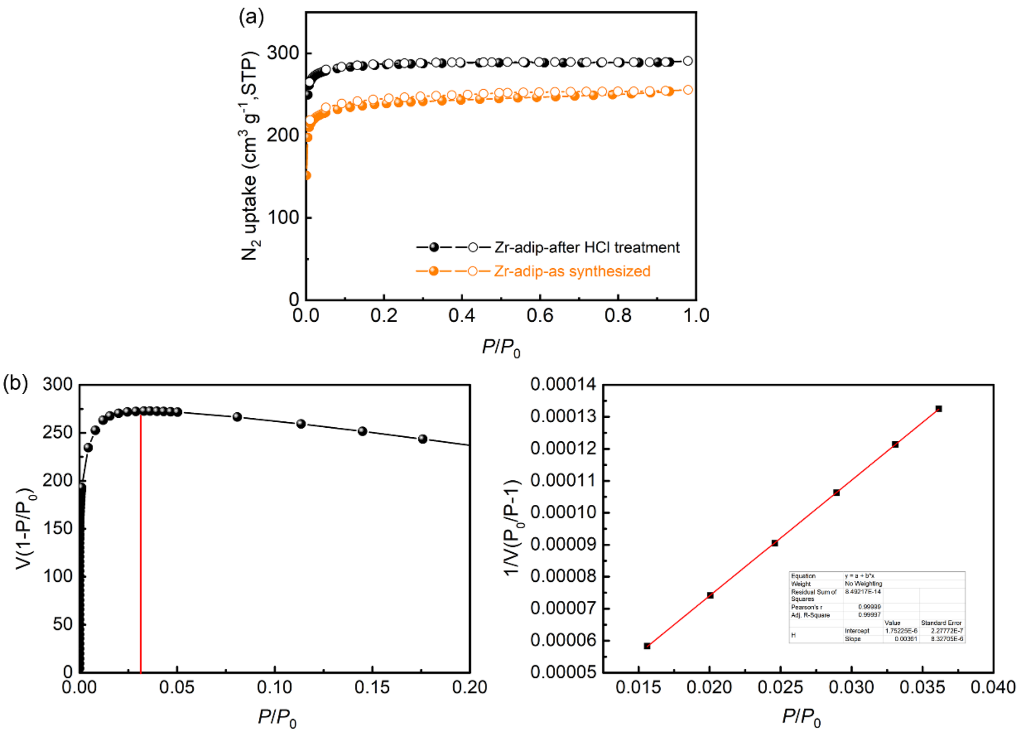

Figure S5. (a) N_2 adsorption isotherms at 77 K for Zr-adip sample after concentrated HCl treatment and the as-synthesized sample, in which HCl treatment leads to an obvious increase on N_2 uptake at 77 K due to the successful replacement of the formate as hydroxyl groups or water molecules. (b) BET specific surface area fitting of the HCl-treated Zr-adip.

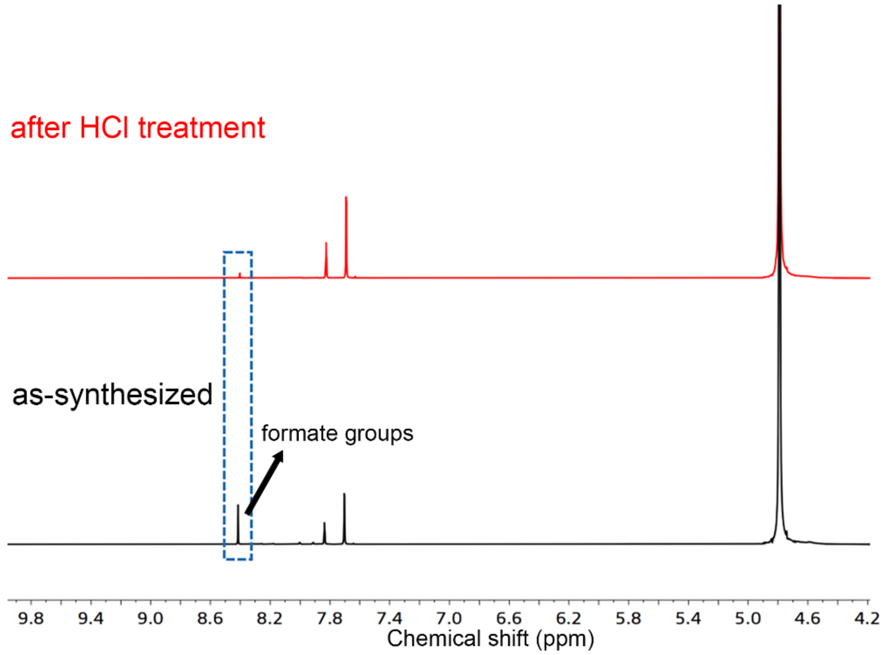

Figure S6. ^1H NMR spectroscopy of as-synthesized (black) and HCl-treated Zr-adip (red) in NaOD/D_2O solution, in which the signal of formate molecule has almost disappeared.

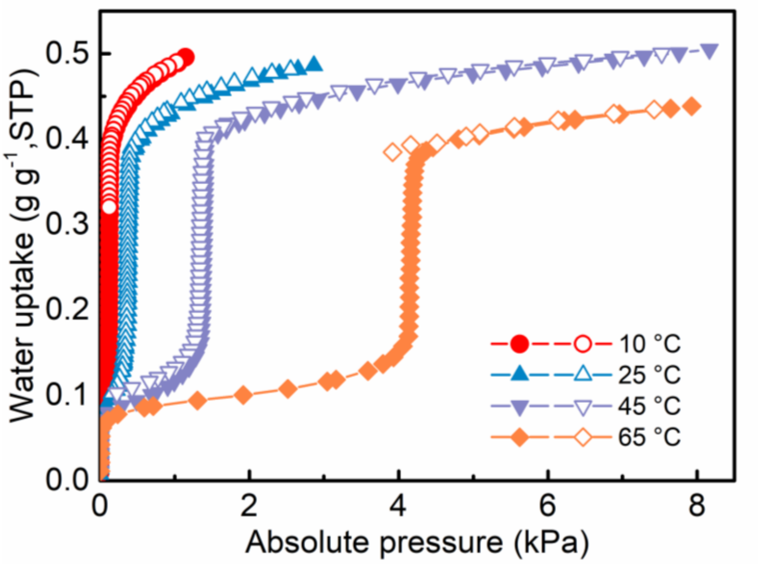

Figure S7. Water adsorption isotherms of Zr-adip at different temperatures under absolute pressure normalized by saturation pressure, solid symbols: adsorption; and open symbols: desorption.

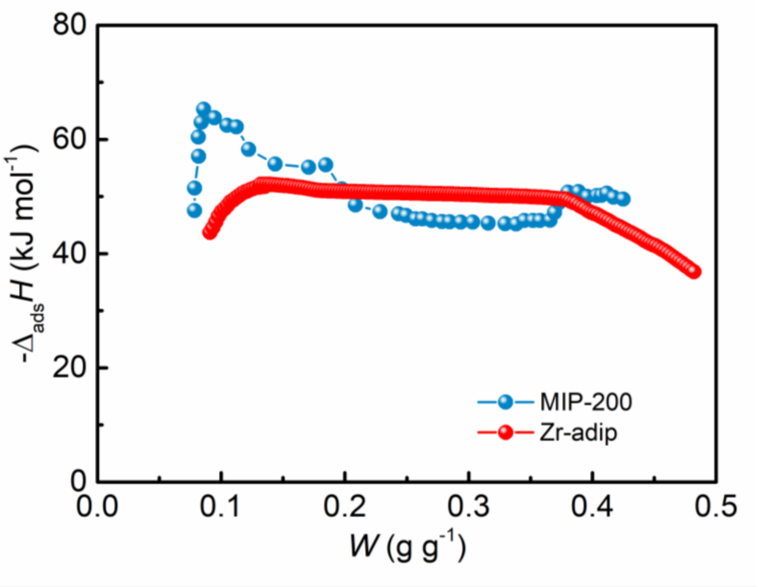

Figure S8. The experimental isosteric heat of water adsorption ($-\Delta_{ads}H$) of Zr-adip (red) calculated by the Clausius–Clapeyron equation, compared with that of MIP-200 (blue) derived from the reported literature.[2]

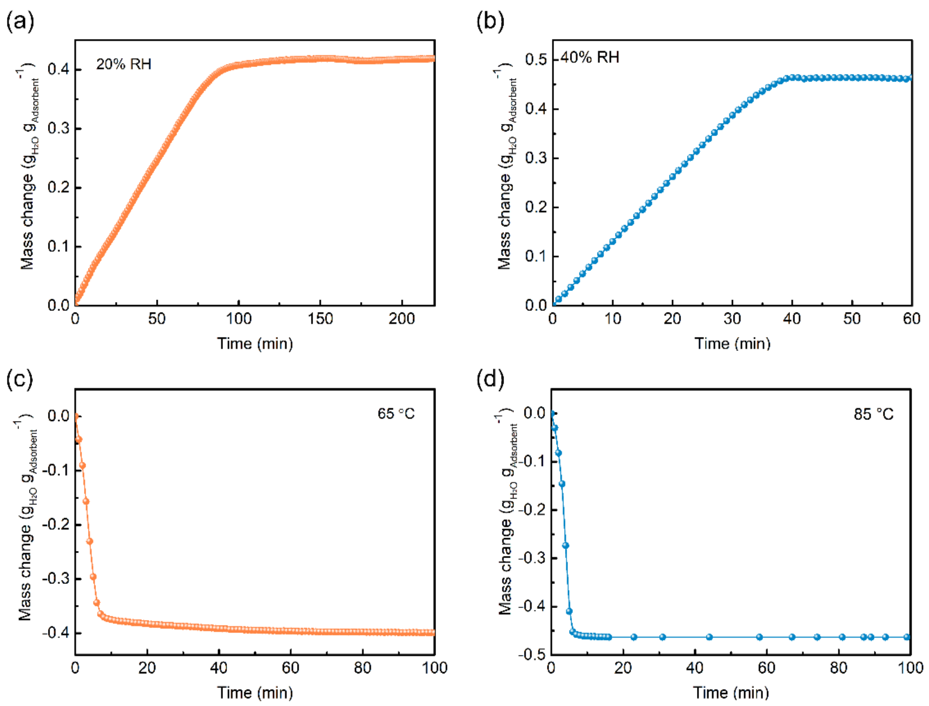

Figure S9. Gravimetric adsorption-desorption profiles of Zr-adip. These profiles were plotted from the steady adsorption-desorption cycle as a function of time, measured by TGA: adsorption at 25 °C and 20% RH (a, top) and desorption at 65 °C/0% RH (c, bottom), and adsorption at 25 °C and 40% RH (b, top) and desorption at 85 °C (d, bottom) under a nitrogen flow (200 mL/min). The sample weight was determined by full dehydration at 120 °C under a dry nitrogen flow (200 mL/min). The ramping rate of desorption temperature is 10 °C/min.

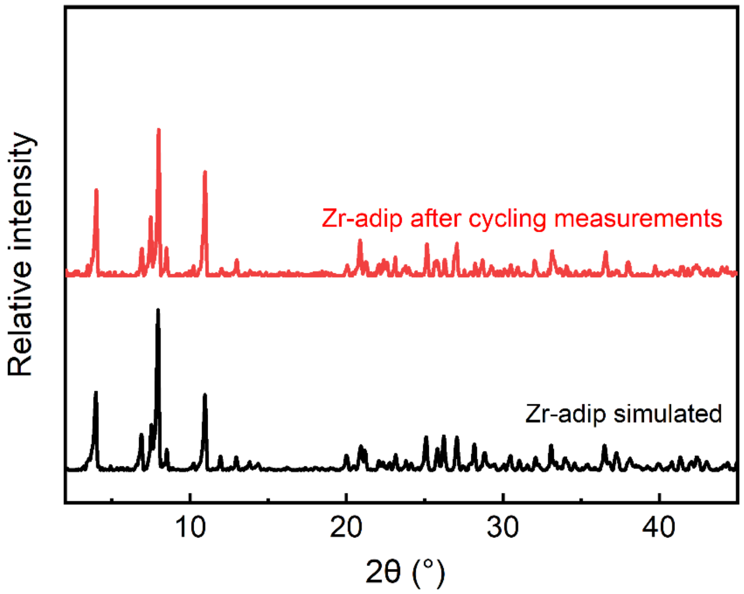

Figure S10. PXRD patterns of Zr-adip after the cycling measurements (red) compared with the simulated XRD patterns from the structures of Zr-adip (black).

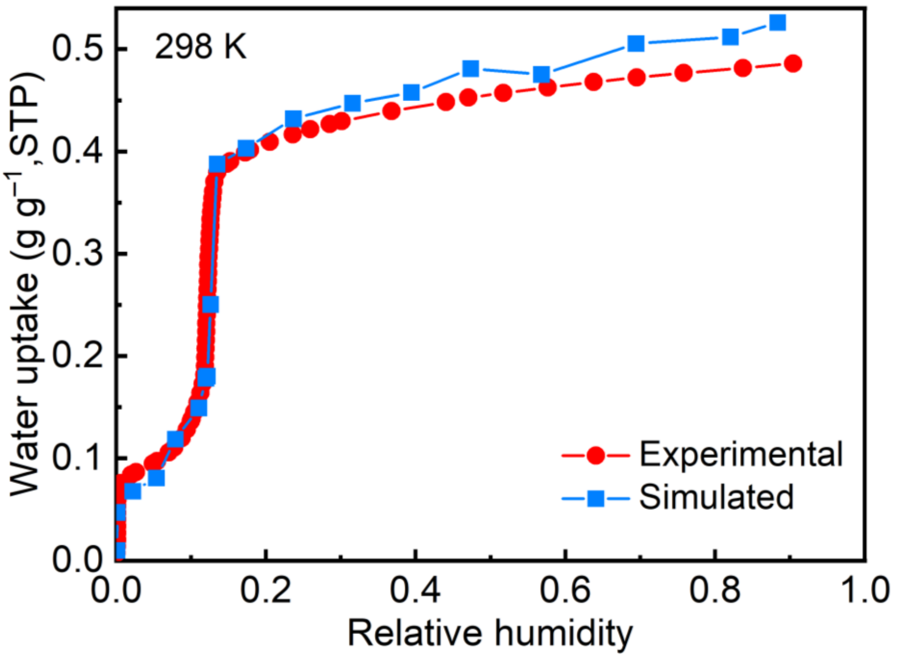

Figure S11. The simulated water adsorption isotherms (blue) and the experimental ones (red) for Zr-adip at 298 K, which are consistent well with each other.

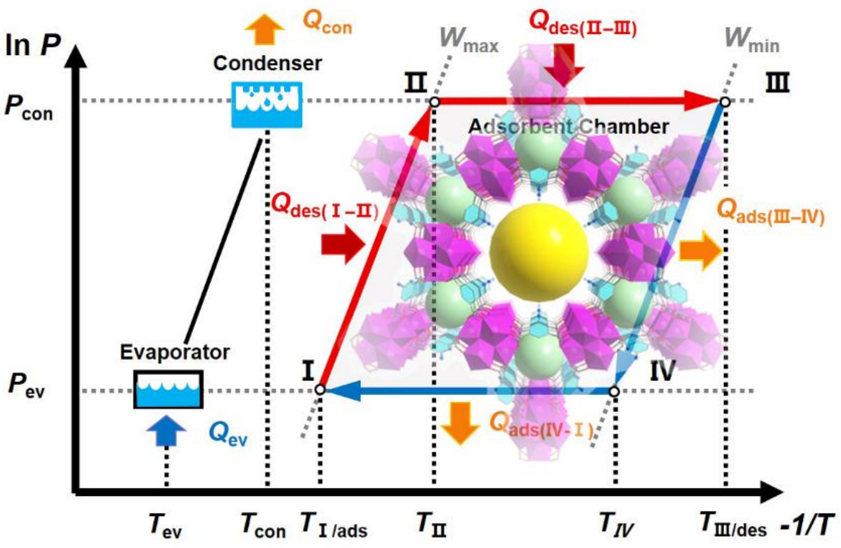

Figure S12. Isosteric cycle diagram of an adsorption heat pump cycle, including the vapor pressure of the chosen working fluid, minimum and maximum isosteres, lines of equal loading, W_{min} and W_{max} (gray dashed lines), temperature and pressure of the evaporator (T_{ev}, P_{ev}) and condenser (T_{con}, P_{con}), desorption temperature (T_{des}), and intermediate cycle temperatures (T_{I}, T_{II}, and T_{IV}). Reproduced with permission.[3] Copyright 2015, American Chemical Society.

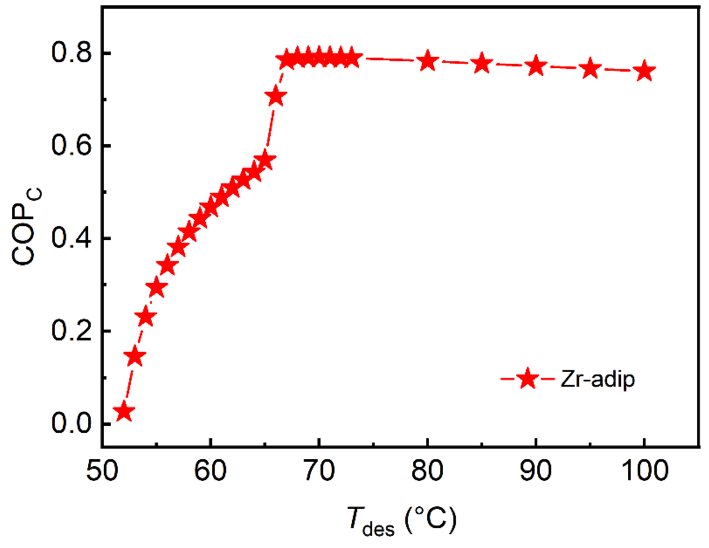

Figure S13. Coefficient of Performance for cooling purposes (COP_C) of Zr-adip as a function of desorption temperature for refrigeration I (T_{ev} = 10 °C, T_{ads} = 30 °C).

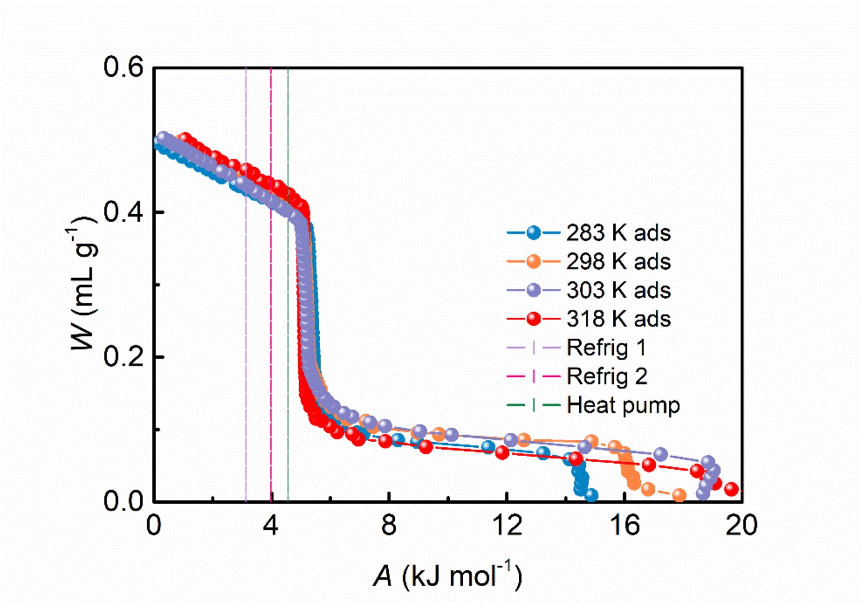

Figure S14. Characteristic curves for water on Zr-adip at different temperatures determined using eq 2. The optimal adsorption potential for refrig-1 cooling (purple), refrig-2 cooling (red), and heat pump heating (green) is indicated by the dashed lines.

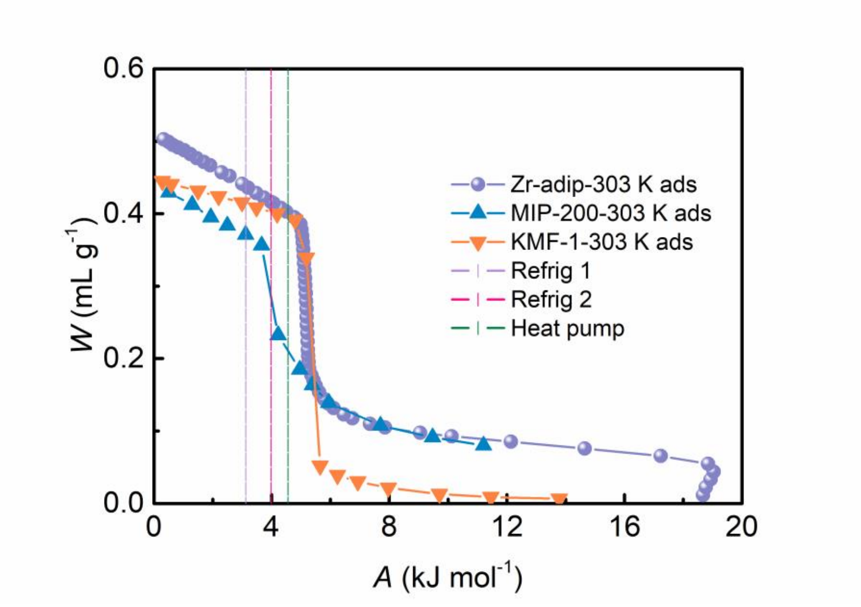

Figure S15. Characteristic curves for water on different materials at 303 K determined by eq 2, using adsorption isotherms from various literature sources. The optimal adsorption potential for refrig-1 cooling (purple), refrig-2 cooling (red), and heat pump heating (green) is indicated by the dashed lines.

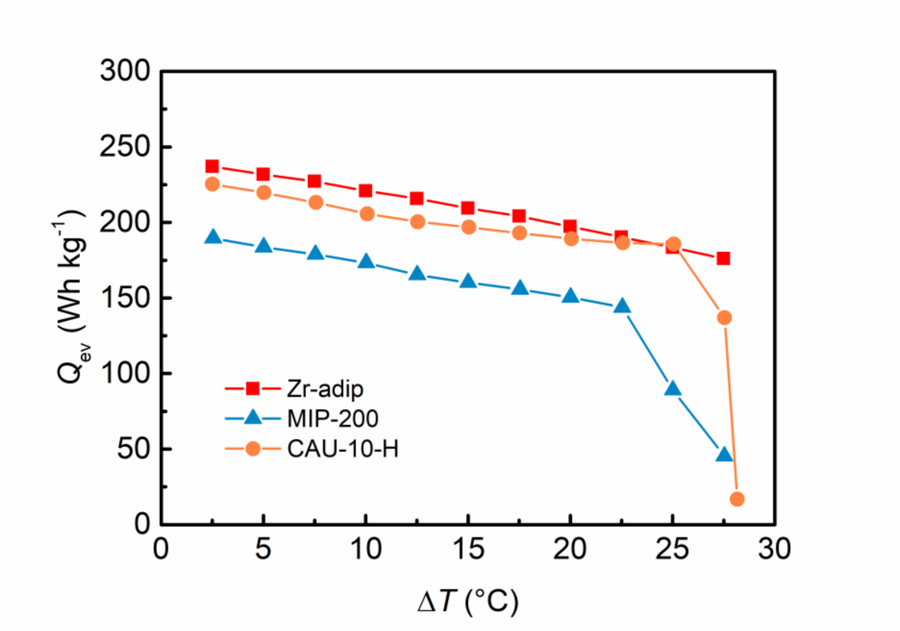

Figure S16. Gravimetric heat transferred from the evaporator in one refrigeration cycle as a function of temperature lift at desorption temperature T_{des} = 70 °C ($\Delta T = T_{con} - T_{ev}$) and T_{con} = 30 °C.

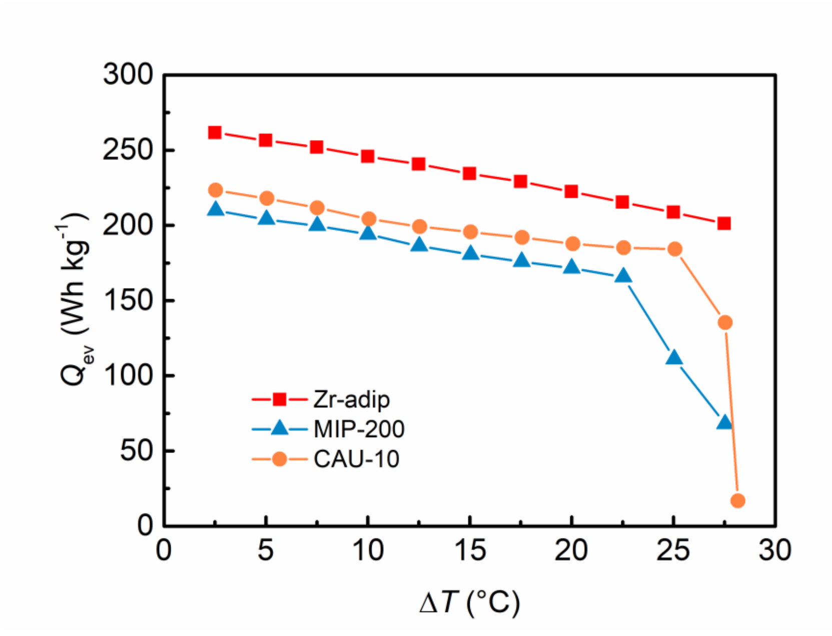

Figure S17. Gravimetric heat transferred from the evaporator in one refrigeration cycle as a function of temperature lift at desorption temperature T_{des} = 80 °C ($\Delta T = T_{con} - T_{ev}$) and T_{con} = 30 °C.

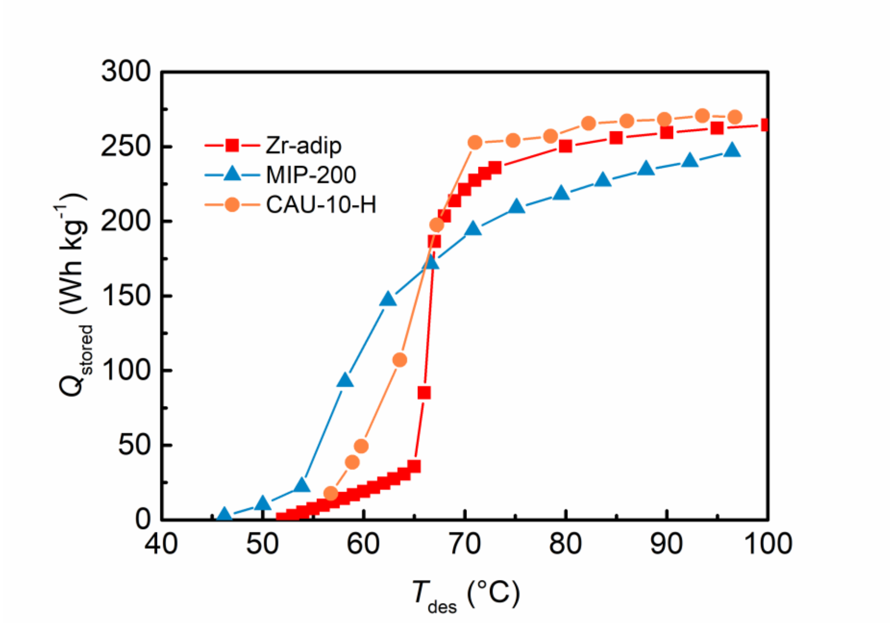

Figure S18. Storable energy capacity as a function of T_{des} for T_{ev} = 10 °C and T_{con} = 30 °C.

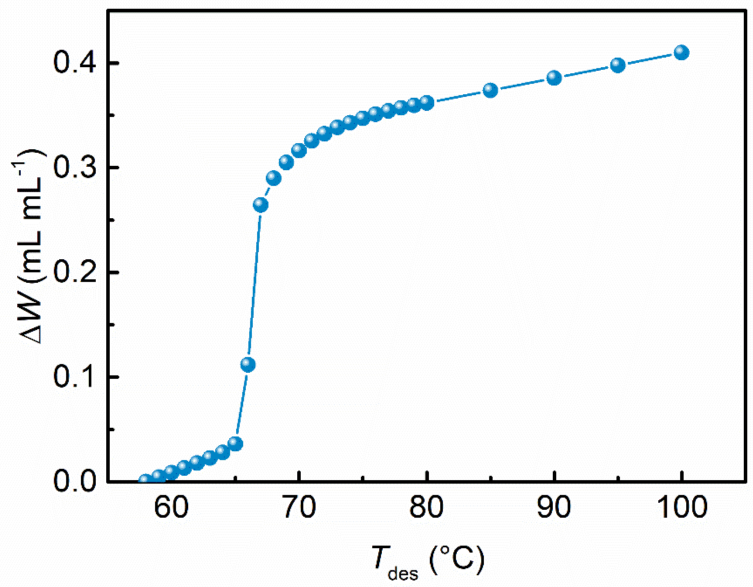

Figure S19. Working capacity (ΔW) as a function of desorption temperature (T_{des}) of Zr-adip for ADCs conditions (T_{ev} = 5 °C, T_{ads} = 30 °C, and T_{cond} = 30 °C).

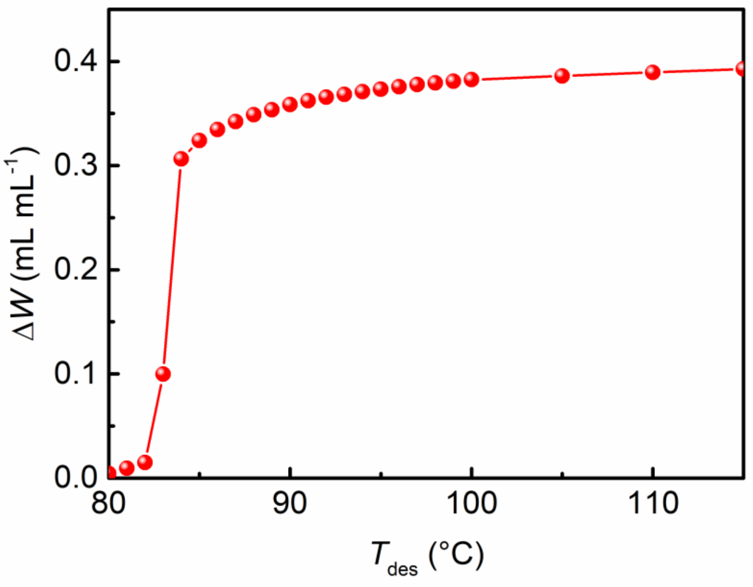

Figure S20. Working capacity (ΔW) as a function of desorption temperature (T_{des}) of Zr-adip for AHP conditions (T_{ev} = 15 °C, T_{ads} = 45 °C, and T_{cond} = 45 °C).

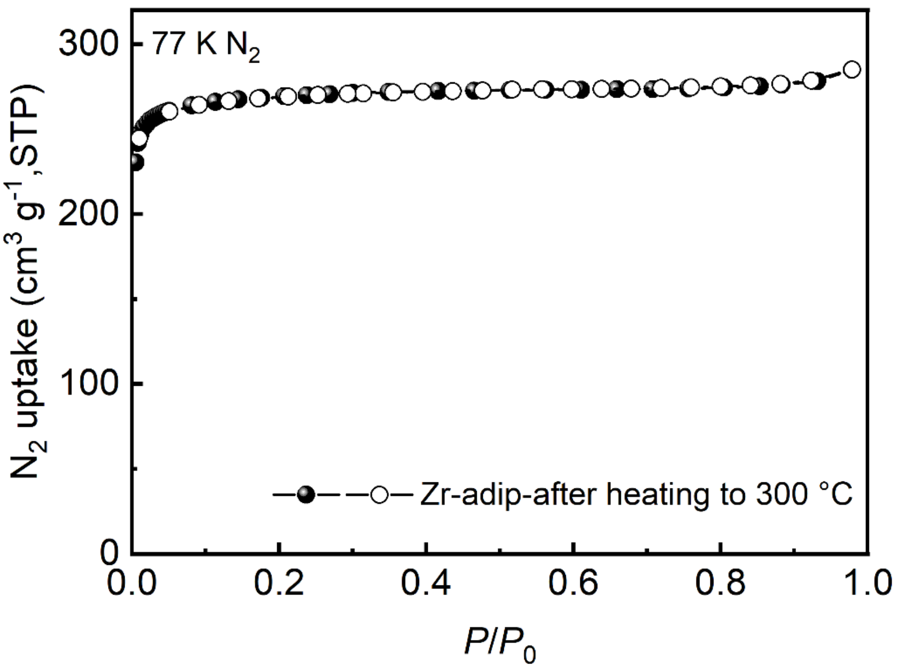

Figure S21. N_2 adsorption isotherms at 77 K for the Zr-adip sample after heating to 300 °C for 12 h, indicating its highly thermal stability.

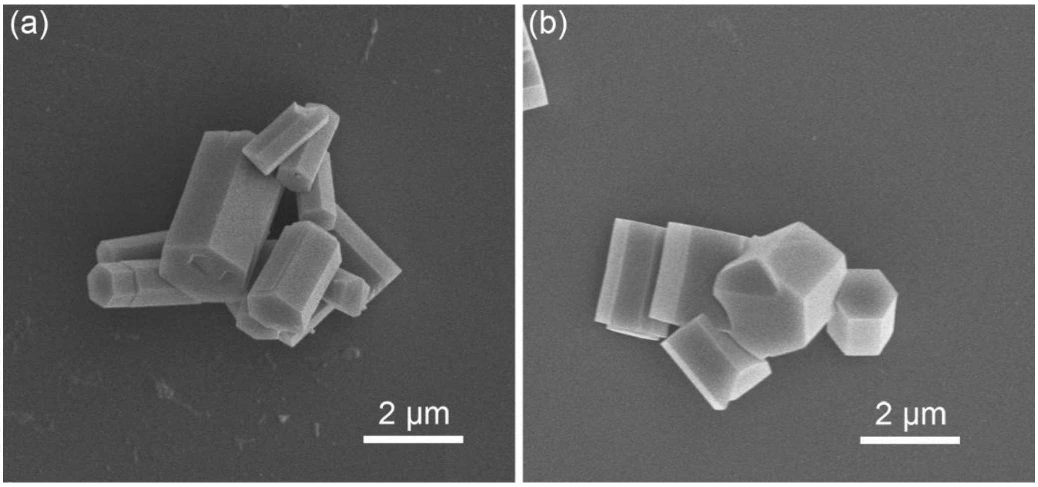

Figure S22. SEM images of (a) as-synthesized samples of Zr-adip and (b) after soaking in water for 2 days.

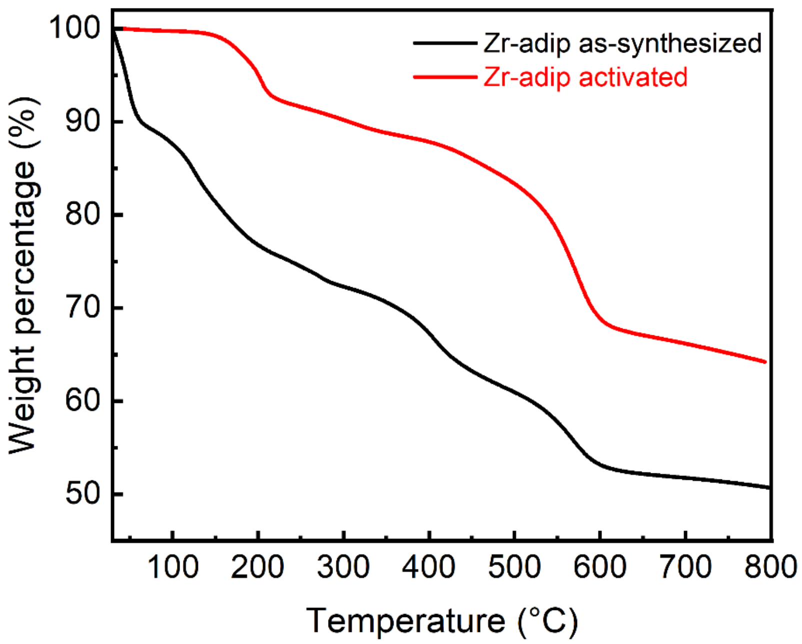

Figure S23. Thermogravimetric analysis profile of as-synthesized (black) and activated samples (red) of Zr-adip. A thermogravimetric analyzer was used to acquire data (TA SDT 650). Protect gas flow rate is 100 mL/min, with a 10 °C/min ramping rate.

References

[1] L. Meng, K. Liu, S. Fu, L. Wang, C. Liang, G. Li, C. Li, Z. Shi, *Journal of Solid State Chemistry*, **2018**, *265*, 285.

[2] S. Wang, J. S. Lee, M. Wahiduzzaman, J. Park, M. Muschi, C. Martineau-Corcos, A. Tissot, K. H. Cho, J. Marrot, W. Shepard, G. Maurin, J.-S. Chang, C. Serre, *Nat. Energy* **2018**, *3*, 985.

[3] M. F. de Lange, K. J. F. M. Verouden, T. J. H. Vlugt, J. Gascon, F. Kapteijn, *Chem. Rev.* **2015**, *115*, 12205.

[4] D. Fröhlich, S. K. Henninger, C. Janiak, *Dalton Trans*. **2014**, *43*, 15300.

[5] D. Lenzen, P. Bendix, H. Reinsch, D. Fröhlich, H. Kummer, M. Möllers, P. P. C. Hügenell, R. Gläser, S. Henninger, N. Stock, *Adv. Mater*. **2018**, *30*, 1705869.

[6] J. S. Lee, J. W. Yoon, P. G. M. Mileo, K. H. Cho, J. Park, K. Kim, H. Kim, M. F. de Lange, F. Kapteijn, G. Maurin, S. M. Humphrey, J.-S. Chang, *ACS Appl. Mater. Interfaces* **2019**, *11*, 25778.

[7] N. Hanikel, M. S. Prévot, F. Fathieh, E. A. Kapustin, H. Lyu, H. Wang, N. J. Diercks, T.

G. Glover, O. M. Yaghi, *ACS Cent. Sci.* **2019**, *5*, 1699.

[8] K. H. Cho, D. D. Borges, U.-H. Lee, J. S. Lee, J. W. Yoon, S. J. Cho, J. Park, W. Lombardo, D. Moon, A. Sapienza, G. Maurin, J.-S. Chang, *Nat. Commun*. **2020**, *11*, 5112.

[9] D. Lenzen, J. Zhao, S.-J. Ernst, M. Wahiduzzaman, A. Ken Inge, D. Fröhlich, H. Xu, H.-J. Bart, C. Janiak, S. Henninger, G. Maurin, X. Zou, N. stock, *Nat. Commun*. **2019**, *10*, 3025.

[10] C. Schlüsener, D. N. Jordan, M. Xhinovci, T. J. Matemb Ma Ntep, A. Schmitz, B. Giesen, C. Janiak, *Dalton Trans*. **2020**, *49*, 7373.

[11] J. J. Oppenheim, J. L. Mancuso, A. M. Wright, A. J. Rieth, C. H. Hendon, M. Dincă, *J. Am. Chem. Soc*. **2021**, *143*, 16343.

[12] H. Furukawa, F. Gándara, Y.-B. Zhang, J. Jiang, W. L. Queen, M. R. Hudson, O. M. Yaghi, *J. Am. Chem. Soc*. **2014**, *136*, 4369.

[13] A. Cadiau, J. S. Lee, D. D. Borges, P. Fabry, T. Devic, M. T. Wharmby, C. Martineau, D. Foucher, F. Taulelle, C.-H. Jun, K. Hwang, N. Stock, M. F. De Lange, F. Kaptejin, J. Gascon, G. Maurin, J.-S. Chang, C. serre, *Adv. Mater*. **2015**, *27*, 4775.

[14] N.Tannert, C. Jansen, S. Nießing, C. Janiak, *Dalton Trans*. **2019**, *48*, 2967.

[15] A. Freni, L. Bonaccorsi, L. Calabrese, A. Caprì, A. Frazzica, A. Sapienza, *Appl. Therm. Eng*. **2015**, *82*, 1.

[16] F. Jeremias, V. Lozan, S. K. Henninger, C. Janiak, *Dalton Trans*. **2013**, *42*, 15967.
